# The Irish Potato Famine Pathogen *Phytophthora infestans* Translocates the CRN8 Kinase into Host Plant Cells

**DOI:** 10.1371/journal.ppat.1002875

**Published:** 2012-08-23

**Authors:** Mireille van Damme, Tolga O. Bozkurt, Cahid Cakir, Sebastian Schornack, Jan Sklenar, Alexandra M. E. Jones, Sophien Kamoun

**Affiliations:** 1 The Sainsbury Laboratory, Norwich Research Park, Norwich, United Kingdom; 2 Laboratory of Phytopathology, Wageningen University, Wageningen, The Netherlands; 3 United States Department of Agriculture-Agricultural Research Service, The Plant Stress and Germplasm Development Unit, Lubbock, Texas, United States of America; University of Melbourne, Australia

## Abstract

Phytopathogenic oomycetes, such as *Phytophthora infestans*, secrete an arsenal of effector proteins that modulate plant innate immunity to enable infection. We describe CRN8, a host-translocated effector of *P. infestans* that has kinase activity *in planta*. CRN8 is a modular protein of the CRN effector family. The C-terminus of CRN8 localizes to the host nucleus and triggers cell death when the protein is expressed *in planta*. Cell death induction by CRN8 is dependent on its localization to the plant nucleus, which requires a functional nuclear localization signal (NLS). The C-terminal sequence of CRN8 has similarity to a serine/threonine RD kinase domain. We demonstrated that CRN8 is a functional RD kinase and that its auto-phosphorylation is dependent on an intact catalytic site. Co-immunoprecipitation experiments revealed that CRN8 forms a dimer or multimer. Heterologous expression of CRN8 *in planta* resulted in enhanced virulence by *P. infestans*. In contrast, *in planta* expression of the dominant-negative CRN8^R469A;D470A^ resulted in reduced *P. infestans* infection, further implicating CRN8 in virulence. Overall, our results indicate that similar to animal parasites, plant pathogens also translocate biochemically active kinase effectors inside host cells.

## Author Summary

*Phytophthora infestans* is the causal agent of late blight on potato and tomato. It is now well established that oomycetes, such as *P. infestans*, secrete an arsenal of effector proteins that modulate plant innate immunity to enable infection. The Crinkler (CRN) effector family containing the LFLAK translocation motif represents a new class of cytoplasmic effectors from oomycetes. The N-terminal domain, which includes this motif, is dispensable for cell death induction *in planta*, indicating the presence of modular domains that are involved in distinct processes. The C-terminal/effector domain of CRN8 localizes to the host nucleus and nuclear localization is required for triggering cell death. The CRN8 effector domain has similarity to plant Serine/Threonine RD kinases. This intriguing finding raises the possibility that CRN8 mimics a specific class of plant enzymes, a feature that has been observed for several other pathogen effectors. Using biochemical assays, we confirmed that the C-terminal domain of CRN8 has kinase activity, and have identified by mass spectrometry five serines shown to be auto-phosphorylated. To the best of our knowledge, CRN8 is the first reported secreted active kinase from a microbial plant pathogen.

## Introduction

Phytopathogenic oomycetes, such as *Phytophthora* spp., cause some of the most destructive plant diseases in the world [1]. *Phytophthora* spp. are hemibiotrophic pathogens, meaning that they have a two-step infection process: an early biotrophic phase (the first 2–3 days after infection), followed by a second phase characterized by extensive host tissue necrosis which enables additional growth and sporulation of the pathogen [2]. To achieve this level of host colonization, plant and animal pathogens secrete molecules, termed effectors, that interfere with host immune pathways and enable host colonization [3]–[5]. Oomycetes secrete hundreds of effector proteins that are either translocated inside host cells or act in the apoplast [6], [7]. Although some of the apoplastic effectors are inhibitors of plant hydrolases, the majority of oomycete cytoplasmic (host-translocated) effectors, namely members of the RXLR and Crinkler (CRN) families, lack similarity to known proteins and their biochemical activities remain largely unknown [6], [8]–[10]. Therefore, elucidating the molecular mechanisms underlying effector activity remains challenging and is dependent on identifying host targets of these effectors [10], [11].

In the case of bacterial plant pathogens, much has been learned from studying the role of effectors that target various host processes once delivered inside host cells. Several of these bacterial effectors, such as the protein tyrosine phosphatase HopPtoD2/HopAO1 [12], [13] and the phosphothreonine lyases OspF and HopAI [14], [15], are enzymes that alter host immunity by targeting signaling components such as mitogen-activated protein kinases (MAPKs) [3], [16]. However, most effectors of filamentous eukaryotic pathogens (oomycetes and fungi) lack similarity to known enzymes or proteins [4], [8], making functional predictions nearly impossible. One notable exception is the host-translocated metalloprotease AvrPita of the rice blast pathogen *Magnaporthe oryzae* [17]. More recently, Avr3b, a *Phytophthora sojae* RXLR effector, was shown to carry a domain with similarity to Nudix hydrolases [18]. The Nudix motif is important for AVR3b virulence function but is not required for activation of the resistance protein Rps3b [18], [19]. This report focuses on CRN8, a host-translocated effector of *Phytophthora infestans* that has similarity to serine/threonine kinases and is a candidate enzyme effector.

Even though functional secreted kinases have been described in animal pathogens, this class of effectors has not been reported for plant pathogens to date. Several animal pathogen kinases translocate inside host cells and perturb various host cell processes [20], [21]. The bacterium *Yersinia pestis*, the causal agent of the bubonic plague, secretes the virulence determinant kinase YpkA into eukaryotic host cells, which eventually leads to alterations in cell morphology [22], [23]. *Toxoplasma gondii*, an obligate intracellular parasite that causes toxoplasmosis, secretes kinases that are injected from secretory parasitic organelles (rhoptries) into the host cell [24]. Recent studies have shown that certain rhoptry proteins (ROPs), specifically the tandem cluster of polymorphic ROP5 pseudokinases, are essential for pathogen virulence [25], [26]. Other ROPs, for instance the kinases ROP16 and ROP18, have been previously shown to be indispensable for full virulence [27], [28]. ROP16 is secreted into the host and is redirected to the nucleus [29] where it phosphorylates the host proteins Signal Transducer and Activator of Transcription-3 (STAT3) [30] and STAT6 [31], leading to altered cytokine profiles and repression of IL-12 signaling [29]. ROP18, an active kinase that trans-phosphorylates immunity related GTPases that plays a major role in *T. gondii* proliferation [32]–[34]. The kinase activity of ROP18 also is essential for proteasome-dependent degradation of activating transcription factor 6 (ATF6β) [35].

The CRNs form a major class of oomycete cytoplasmic effectors that are known to alter host responses [4], [36], [37]. They were originally identified following an *in planta* expression screen of candidate secreted proteins of *P. infestans* [36]. Intracellular expression of several CRN C-termini in plants results in plant cell death and induction of defense-related genes [9], [36]. Therefore, the CRNs appear to perturb host cellular processes similar to many plant pathogen effectors, causing macroscopic phenotypes such as cell death, chlorosis, and tissue browning when expressed in host cells [36], [38]–[40]. More recently, CRN N-termini were shown to be functionally interchangeable with the N-termini of RXLR effectors, and to successfully deliver the C-terminal portion of the RXLR effector protein AVR3a inside plant cells [41]. Therefore, CRN effectors are modular proteins consisting of conserved N-termini required for translocation inside host cells and highly diverse C-termini responsible for the effector's biochemical activity [9], [33], [41], [42]. Interestingly, the CRNs appear to be chimeric, with strong evidence of recombination after the HVLVXXP motif that occurs at the end of the DWL domain just prior to the C-terminus [9]. The C-termini of CRN proteins are diverse and typically localize to plant nucleus [42]. Specifically, CRN8 requires a functional nuclear localization signal (NLS) for nuclear accumulation and cell death induction [42].

The D2 domain of CRN8 has significant similarity to protein serine/threonine kinases of the RD class [9]. RD kinases are defined as those kinases in which the conserved catalytic aspartate is preceded by an arginine residue in the kinase subdomain VI [43]. RD kinases are regulated by activation loop phosphorylation [9], [43]. Serine/threonine kinases phosphorylate the OH group of serine or threonine, while other kinases act on tyrosine, and a number (dual-specificity kinases) act on all three [44]. Protein kinases are one of the largest groups of kinases. By adding phosphate groups to substrate proteins, kinases direct the activity, localization, and overall function of many proteins, ultimately regulating almost all cellular processes. Dimerization has a crucial role in the regulation of many kinases and, as is the case with MAP kinases, can have a profound impact on their regulation and substrate selectivity [45]. Kinase activation by dimerization forms another layer of regulation. It can be exploited to interfere with normal kinase activity using dominant-negative kinase inactive mutants. For example, in cancer research the mammalian serine/threonine kinase Akt is an important regulator of cell survival and cell proliferation. However, co-expression of a kinase-inactive mutant of Akt in cells interfered with active Akt and inhibited cell proliferation and apoptotic response in tumor cells [46]. Another example is the expression of the catalytically inactive tyrosine phosphatase, HopAO1, that has a dominant-negative effect on the function of the wild type HopAO1 in infected plant cells [13].

In this study, we characterized CRN8, a secreted serine/threonine kinase of *P. infestans*. The predicted catalytic domain of CRN8 includes amino acid sequence 454 to 573 with a conserved catalytic aspartate (D 470) adjacent to an arginine (R 469). Here, we demonstrated that CRN8 is an active *P. infestans* kinase that displays catalytic activity and is auto-phosphorylated inside plant cells. Transient *in planta* over-expression of CRN8 resulted in cell death, and at least five phosphorylated serines were identified in CRN8 by mass spectrometry. Substitution of all five serines into alanines resulted in loss of cell death activity. Transient co-expression of wild type CRN8 and a kinase-inactive CRN8 mutant in *Nicotiana benthamiana* resulted in a large reduction of CRN8 cell death activity consistent with dominant negative effects. We also discovered that CRN8 is able to form dimers *in planta*. Finally, we showed that *in planta* expression of CRN8 enhances *P. infestans* virulence, whereas expression of the dominant-negative mutant resulted in reduced virulence.

## Results

### Expansion of the CRN8 family in *P. infestans*

**Figure 1:**
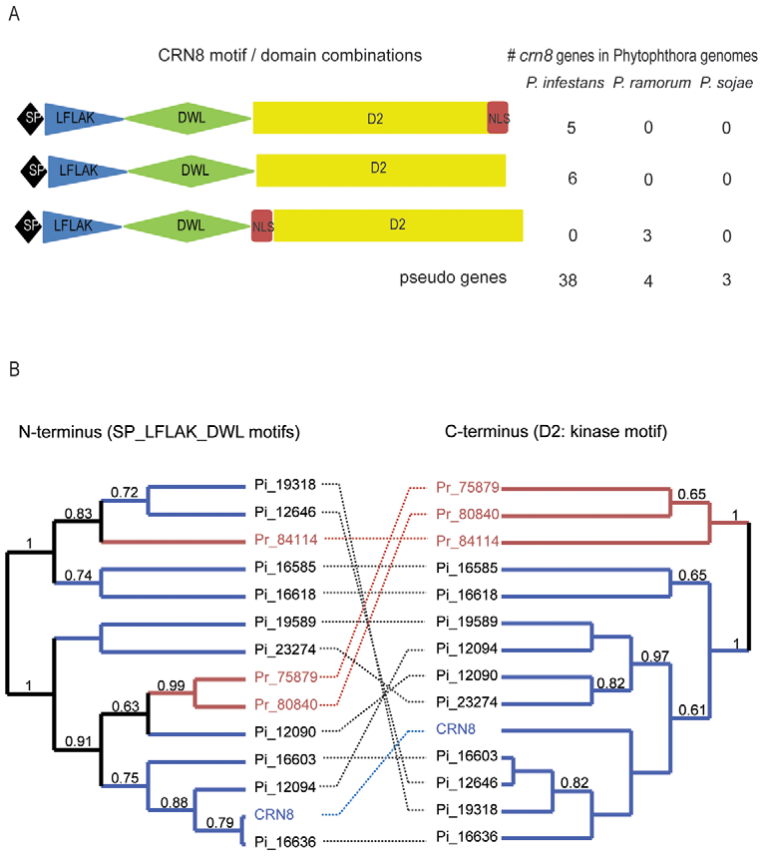
CRN8 domain composition and phylogenetic analysis of CRN8 paralogs. (A) Cartoon of the three different motif combinations of CRN8 orthologs present in three sequenced *Phytophthora* genomes. The signal peptide (SP, black), LFLAK motif (blue), DWL motif (green), the NLS domain (red), and the kinase-homologous D2 domain (yellow) are presented as separate building blocks. The copy number of CRN8 paralogs and pseudogenes for three different sequenced *Phytophthora* genomes is stated alongside each CNR8 archetype. (B) Two phylogenetic trees of *P. infestans* (blue) and *P. ramorum* (orange) CRN8 paralogs based on amino acid sequence; the left side shows the N-terminus and the right side indicates the C-terminus. The originally identified CRN8 sequence from *P. infestans* isolate H88069 was also included (named CRN8). The other sequences are presented by their genome number. The N-terminal amino acid sequences in the left tree are connected by a dashed line to the C-terminal amino acid sequences of the right tree.

CRN8 consists of four different conserved domains: the signal peptide (SP), the LFLAK motif, the DWL domain, and the D2 domain, which shows homology to the RD class of protein kinases (Figure 1A) [9]. Based on this domain compilation, ten functional CRN8 paralogs were predicted from the sequenced genome of *P. infestans* isolate T30-4, compared to three in *P. ramorum*, and none in *P. sojae* [9]. Five of the ten *P. infestans* CRN8 paralogs contain a predicted nuclear localization signal (NLS: amino acid sequence KGVRKKHRRA) which occurs after the D2 domain. The three *P. ramorum* CRN8 paralogs also have a predicted NLS (amino acid sequence: KRKRK), however, unlike the *P. infestans* CRN8 paralogs, their NLS sequence occurs prior to the D2 domain (Figure 1A). ClustalW alignments of the D2 domain sequences, the original CRN8 allele from *P. infestans* isolate 88069 [36], the 10 CRN8 paralogs, and the three *P. ramorum* paralogs revealed conservation of the RD kinase catalytic site (marked by the two asterisks) [9]. Nevertheless, the *P. ramorum* CRN8 paralog sequences share only 33% amino acid identity to CRN8 compared to 97–99% identity among the *P. infestans* kinase domains (Figure S1). Analysis of the *Phytophthora* genome sequences also revealed 38 CRN8 pseudogenes in *P. infestans*, four in *P. ramorum* and three from *P. sojae* (Figure 1A). The high number of *P. infestans* CRN8 paralogs, the presence of many pseudogenes, and the high amino acid conservation is an indication that the CRN8 family in *P. infestans* arose from a recent expansion.

The modular domain structure of the CRNs [9] implies that each domain may evolve separately. Taking this into account, we examined the phylogenetic relationship among CRN8 paralogs by analyzing the C-terminal amino acid sequence (the D2 domain) separately from the N-terminal sequence (including the SP, LFLAK and DWL domains). Figure 1 illustrates the two separate phylogenetic trees of the *P. infestans* and *P. ramorum* CRN8 paralogs. The N-terminal amino acid sequences in the left tree are connected to their corresponding C-terminal amino acid sequence in the right tree by a dashed line. In the N-terminal phylogenetic tree, the *P. ramorum* CRN8 sequences are present in different clades, yet analysis of the C-terminus shows *P. ramorum* D2 sequences to be grouped into a single clade when compared to *P. infestans* D2 sequences. From these phylogenetic analyses, we conclude that both termini evolved separately. The separate clustering of *P. infestans* and *P. ramorum* D2 domains suggests that the expansion of the CRN8 family in *P. infestans* occurred after the divergence of *P. infestans* and *P. ramorum*. This feature of CRN8, together with the phylogenetic studies on all *P. infestans* CRNs [9], is indicative of recombination between different CRN domains and points to a mechanism responsible for increasing CRN diversity. Expansion of the *P. infestans* CRN8 family and conservation of its D2 kinase domain suggests that CRN8 kinase activity was preserved in the *P. infestans* lineage.

### The D2 kinase domain of CRN8 causes cell death *in planta*

**Figure 2:**
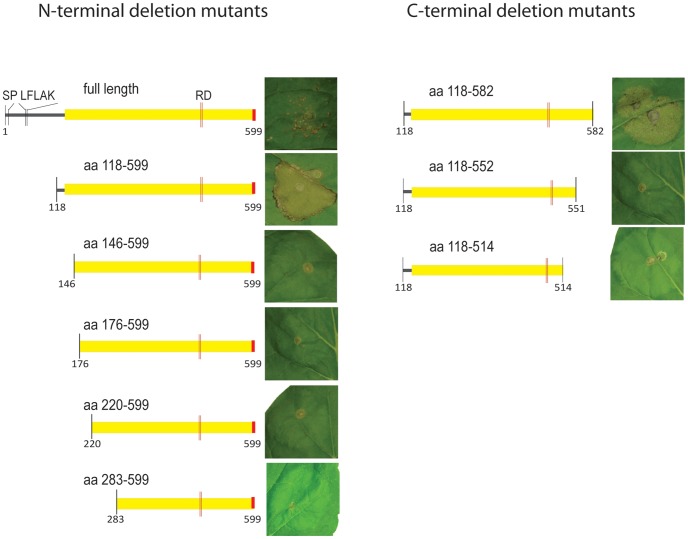
Defining the CRN8 cell death induction domain. *In planta* expression of various N-terminal or C-terminal deletions of CRN8, demonstrating the minimal domain necessary for cell death induction. The D2 domain is indicated in yellow, whereas the red portion indicates the position of the functional NLS.

The C-terminus of CRN8 was previously shown to induce cell death when expressed *in planta* [9]. To further define the domain of CRN8 that is required for cell death induction, we used *A. tumefaciens*-mediated transient expression to assay five N-terminal and three C-terminal deletion mutants for cell death activation in *N. benthamiana* (Figure 2). The only N-terminal mutant that retained the ability to give a strong cell death induction was the smallest deletion, consisting of amino acid residues 118 to 599 (Figure 2). For C-terminal deletion mutants, cell death induction was partially lost in the 118–582 deletion mutant, and completely lost in any of the larger deletions, indicating that an intact C-terminus containing amino acids 118 to 599 is required for full activity (Figure 2). These results indicate that the region required for cell death induction includes the entire kinase domain, as well as the C-terminal NLS; a finding consistent with previous reports [42]. The full-length CRN8 protein with signal peptide, caused cell death but at weaker levels than the C-terminal domain alone (Figure 2). Previous studies with oomycete cytoplasmic effectors have demonstrated stronger phenotypes when effectors are expressed *in planta* without secretion signals [41], [47], [48]. The presence of the signal peptide could result in miss-targeting the protein from the endoplasmic reticulum to the cytoplasm or re-entry following secretion [41], [47], [48]. From the deletion mutant analysis, we conclude that the whole D2 domain and the C-terminal NLS are required for cell death induction.

### CRN8 has kinase activity *in planta*

**Figure 3:**
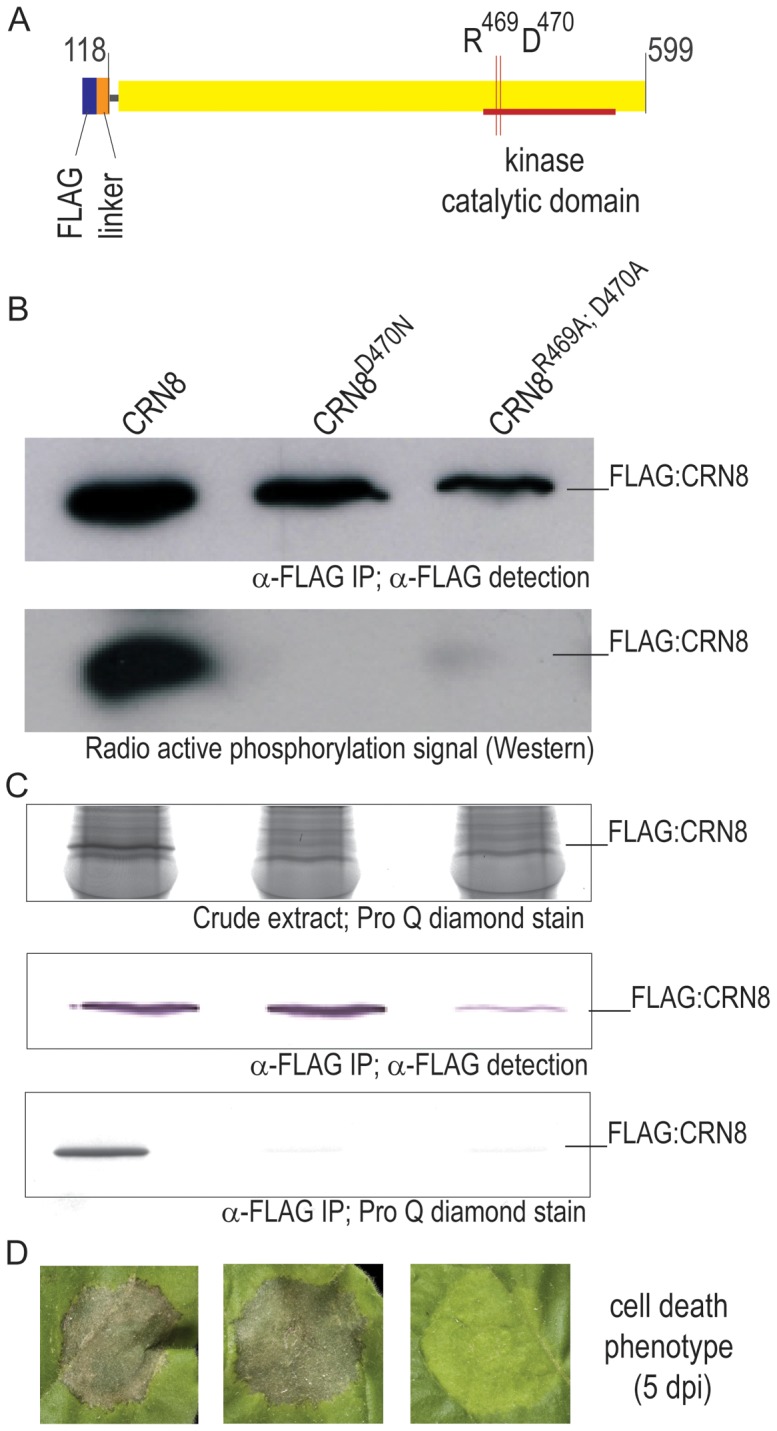
CRN8 is an active kinase *in planta*. (A) An overview of the CRN8 construct, including the catalytic domain (red) and the amino acid positions of Arginine (R) at 469 and Aspartate (D) at 470. (B) The upper panel indicates the protein input for the kinase assay. The lower panel shows an auto-radiogram detecting phosphorylation state of CRN8, CRN8^D470N^, and CRN8^R469A;D470A^, with only CRN8 producing a signal. (C) Phosphorylated CRN8 protein was detected in crude plant protein extract (top panel) and in FLAG immuno-purified protein (lower panel) by Pro-Q Diamond phosphoprotein in gel stain. The middle panel shows that all three fusion proteins were present, as detected by FLAG antibody on a Western blot. (D) Macroscopic cell death associated with *A. tumefaciens*-mediated transient expression of FLAG:CRN8 and kinase-inactive mutants FLAG:CRN8^D470N^ and FLAG:CRN8^R469A;D470A^ in *N. benthamiana*. Picture was taken five days post *A. tumefaciens* infiltration in *N. benthamiana*.

To determine if CRN8 is a functional kinase, we tested the phosphorylation ability of the D2 kinase domain *in vitro*. Figure 3A illustrates the used FLAG fused protein structure of the CRN8 effector, mutations were generated at the indicated RD site. Large amounts of FLAG epitope-tagged CRN8 fusion protein (amino acids 118–599) and kinase-inactive mutant proteins, FLAG:CRN8^D470N^ and FLAG:CRN8^R469A;D470A^, were expressed *in planta* and purified by FLAG immuno-precipitation. To verify that the proteins were present, a fraction of each purified protein was subjected to SDS-PAGE and Western blot analysis with FLAG antibody (Figure 3B, upper panel). When these samples were subjected to an *in vitro* kinase assay with γ-P^32^-ATP, an auto-phosphorylated band corresponding to the FLAG:CRN8 (WT) protein was detected, but no signal was detectable for the FLAG:CRN8^D470N^ and FLAG:CRN8^R469A;D470A^ mutant proteins on the autoradiogram (Figure 3B, lower panel). In Figure 3C, we further examined the phosphorylation status of CRN8 and its mutants by using phosphorylation-specific staining (ProQ Diamond [49]) of crude and immuno precipitated material. Only CRN8 (WT) protein was found to be phosphorylated in both crude and immuno-purified plant protein extract (Figure 3C, top panel). While all fusion proteins were present at detectable levels (Figure 3C, middle panel), only the FLAG:CRN8 (WT) protein produced a signal when incubated with the ProQ Diamond stain [49] (Figure 3C, bottom panel). We conclude that CRN8 is an active kinase capable of auto-phosphorylation.

### Kinase activity is not required for cell death induction

To test if the kinase activity of the CRN8 D2 domain is required for cell death induction, we expressed FLAG:CRN8 (WT), FLAG:CRN8^D470N^ and FLAG:CRN8^R469A;D470A^ in *N. benthamiana* by *Agrobacterium tumefaciens*-mediated transient expression. Macroscopic cell death was visible five days post expression for FLAG:CRN8 (WT) and FLAG:CRN8^D470N^ proteins, but not for the FLAG:CRN8^R469A;D470A^ protein (Figure 3D). The observation that the CRN8^D470N^ inactive-kinase mutant still causes cell death, suggests that kinase activity is not required for CRN8-induced cell death.

### Five phosphorylated serines in CRN8

**Figure 4:**
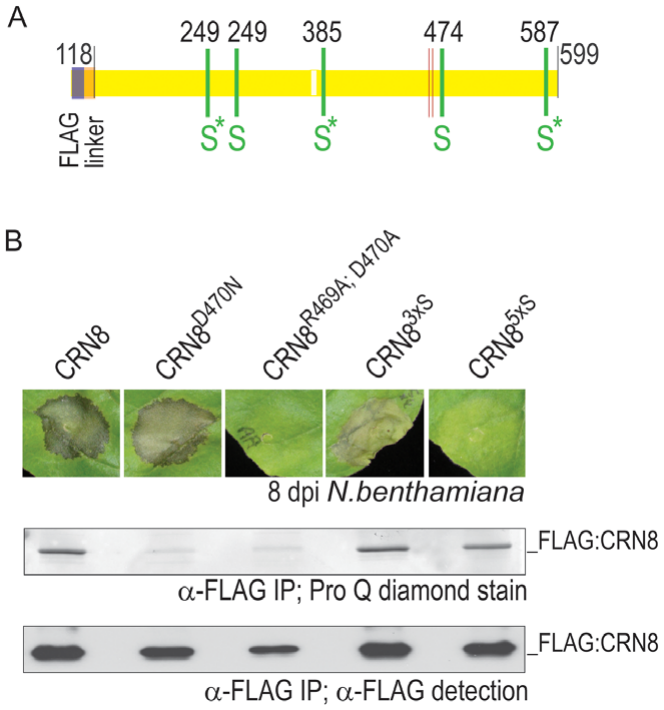
Identification and role of phosphorylated serines in CRN8 cell death induction. (A) A cartoon of the CRN8 protein (yellow) with the five phosphorylated serines identified by LC MS/MS denoted by green bars. Portion of CRN8 shown in light yellow was not represented in peptide coverage. Asterisks indicate serines that were included in the CRN8^S3xA^ triple mutant. FLAG sequence (blue portion) and the linker sequence (orange portion) are included. (B) The top panel shows macroscopic cell death associated with *A. tumefaciens*-mediated transient expression of FLAG:CRN8, FLAG:CRN8^D470N^, FLAG:CRN8^R469A;D470A^, FLAG:CRN8^S3xA^, and FLAG:CRN8^S5xA^ in *N. benthamiana*. Pictures were taken 8 days after inoculation. The middle panel shows that phosphorylated protein was detected for FLAG:CRN8, FLAG:CRN8^S3xA^, and FLAG:CRN8^S5xA^ the in FLAG immuno-purified protein by Pro-Q Diamond phosphoprotein in gel stain. The bottom panel shows that all five FLAG immuno-purified fusion proteins were present, as detected by FLAG antibody on a Western blot.

To identify phosphorylation sites in CRN8, we purified FLAG and GFP epitope-tagged CRN8 wild type (amino acids 118–599) expressed *in planta*. Appropriate bands from immuno-purified samples were excised after SDS-PAGE separation and trypsin-digested before submitting for LC-MS/MS (LTQ-Orbitrap). From the analyzed samples we obtained 99% coverage of the CRN8 protein (Figure 4A, light yellow box is an area not observed in the MS data), and identified five phosphorylated serines in the CRN8 protein. Serines at amino acid position 249, 281, 385, 474 and 587 (indicated by the green bars in Figure 4A displayed in the FLAG tagged construct) were phosphorylated. Annotated spectra for each identified phosphorylated serine are provided in Dataset S1 and their corresponding Mascot scores in Table S1.

### Substitution of phosphorylated serines into alanines results in decreased cell death induction

To determine the relevance of the phosphorylated serines for both kinase activity and cell death induction we substituted three (Figure 4A, indicated by an asterisk) or all five of the phosphorylated serines in alanine, thereby generating triple and quintuple serine to alanine FLAG:CRN8 mutant proteins. These mutants, along with FLAG:CRN8 (WT), FLAG:CRN8^D470N^, and FLAG:CRN8^R469A;D470A^, were expressed *in planta* and purified as described above. Phosphorylation levels were determined using the ProQ Diamond stain [49], and revealed phosphorylation of FLAG:CRN8(WT), FLAG:CRN8^S248,385,585A^, and FLAG:CRN8^S249,281,385,474,587A^ proteins (Figure 4B, middle panel). As demonstrated previously (Figure 3C), no phosphorylation was detected in the FLAG:CRN8^D470N^ and FLAG:CRN8^R469A;D470A^ protein samples (middle panel Figure 4B). The presence of all proteins was confirmed by Western blot (Figure 4B, lower panel). The finding that our quintuple serine mutant protein was still phosphorylated (middle panel of figure 4B), indicates that other serines or threonines were also targeted for phosphorylation. Indeed, upon analysis of purified FLAG:CRN8^S249,385,587A^ by LC-MS/MS, we detected a phosphorylated serine residue in the linker sequence between the epitope tag and the CRN8 protein sequence (data not shown).

We also determined that, in addition to the kinase-inactive FLAG:CRN8^R469A;D470A^ mutant described above (Figure 3D), FLAG:CRN8^S249,385,587A^ and FLAG:CRN8^S249,281,385,474,587A^ mutants were also altered in cell death induction when expressed in leaves of *N. benthamiana* (Figure 4B, top panel) and *Solanum lycopersicum* (tomato) cultivar Money Maker Cf-0 (Figure S2). Macroscopic cell death was greatly reduced in the CRN8^S249,385,587A^ mutant, whereas no cell death was detectable in the FLAG:CRN8^S249,281,385,474,587A^ mutant (top panel Figure 4B, Figure S2). Our data indicate that the cell death induced by CRN8 is not a direct result of its kinase activity, but rather a consequence of the phosphorylated state of the five identified serine residues in the CRN8 protein.

### The kinase-inactive mutant CRN8^R469A;D470A^ interferes with CRN8-induced cell death

**Figure 5:**
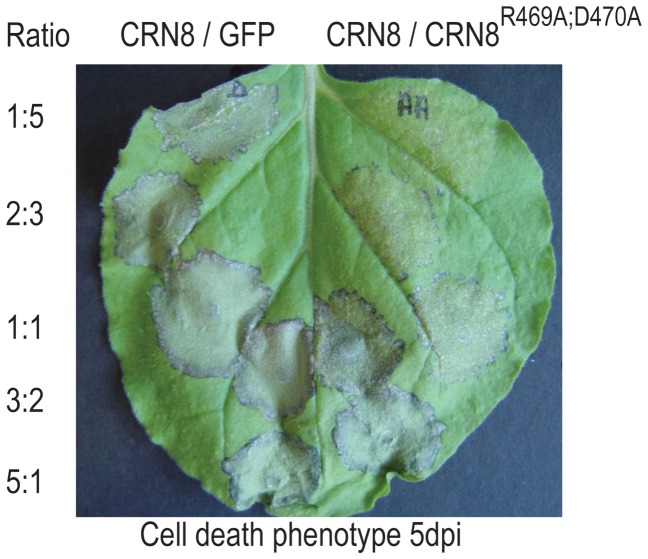
The kinase dead mutant CRN8^R469A;D470A^ suppresses CRN8-induced cell death. *N. benthamiana* leaf displaying CRN8-induced cell death shown five days post infiltration with *A. tumefaciens*. Different inoculation combinations are shown above for each half of the leaf: CRN8 with GFP (left) and CRN8 with CRN8^R469A;D470A^ (right). To the left of the picture, the five different ratios of each tested combination are indicated.

To further explore the cell death induced by CRN8, we tested whether the kinase-inactive FLAG:CRN8^R469A;D470A^ mutant could function in a dominant negative manner to suppress CRN8-induced cell death, as is the case for the HopAO1 bacterial effector [13]. Mixtures of *A. tumefaciens* containing pGR106-CRN8 (amino acids 118–599) and pGR106-CRN8^R469A;D470A^ were infiltrated in various ratios into *N. benthamiana* leaves and scored for their ability to cause cell death. We observed that CRN8-induced cell death was greatly reduced when co-expressed with CRN8^R469A;D470A^, especially in wild type to mutant ratios of 1∶5, 2∶3, and 1∶1 (Figure 5). Samples of CRN8 on the left half of the leaf were co-inoculated with GFP as a negative control and show no effect on CRN8-induced cell death (Figure 5). Cell death suppression by the CRN8^R469A;D470A^ mutant suggests that the inactive kinase has a dominant negative effect on CRN8 cell death induction, and that the inactive kinase mutant acts antagonistically on wild type CRN8.

### The kinase-inactive mutant (CRN8^R469A;D470A^) destabilizes the CRN8 protein

**Figure 6:**
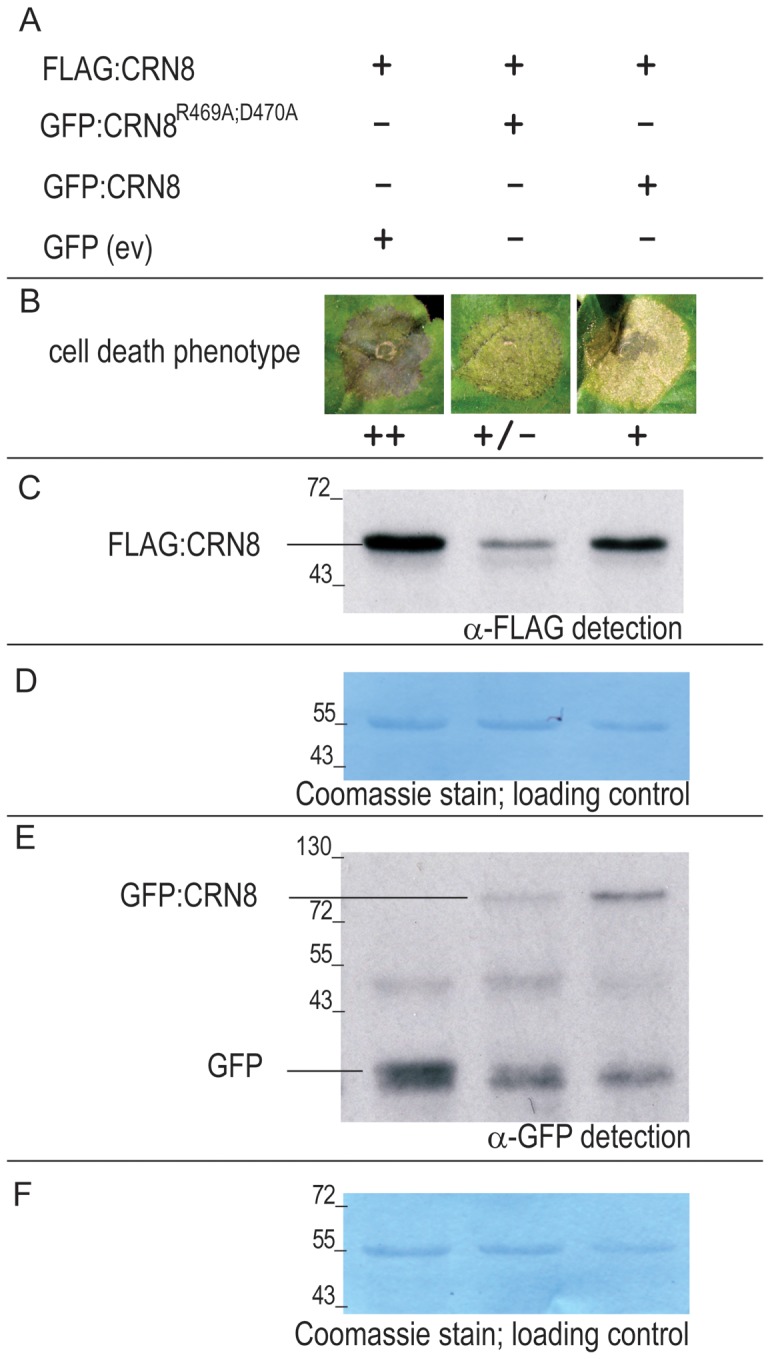
The kinase inactive mutant, CRN8^R469A;D470A^, destabilizes the CRN8 protein. (A) Overview of the different combinations of *Agrobacterium*-mediated co-expressed proteins in *N. benthamiana* leaves. (B) Macroscopic cell death 5 days post infiltration in *N. benthamiana* leaves; strongest cell death classified as ++ and suppressed cell death as +/−. (C) Western blot of FLAG:CRN8 protein inputs probed with FLAG antibody. (D) Coomassie stain indicating equal loading of protein on Western blot depicted in figure 6C. (E) Western blot of GFP, GFP:CRN8, and GFP:CRN8^R469A;D470A^ signals from crude protein extracts probed with GFP antibody. (F) Coomassie stain indicating equal loading of protein on Western blot depicted in figure 6E.

Because co-expression of CRN8^R469A;D470A^ with CRN8 resulted in the suppression of cell death, we tested whether changes in CRN8 protein levels due to CRN8^R469A;D470A^ played a role in this altered phenotype. Combinations of *A. tumefaciens* strains containing FLAG:CRN8, GFP:CRN8, GFP:CRN8^R469A;D470A^, or empty vector GFP (EV:GFP) constructs were infiltrated into *N. benthamiana* leaves and monitored for both cell death induction and protein levels (Figure 6A–E). The first panel of figure 6B shows strong induction of cell death by FLAG:CRN8, indicating that co-expression with the EV:GFP control did not suppress the cell death response. In the second panel, co-expression of FLAG:CRN8 with GFP:CRN8^R469A;D470A^ resulted in a great reduction of CRN8-induced cell death. The third panel shows that cell death occurred when FLAG:CRN8 and GFP:CRN8 were co-expressed, although to a lesser degree than in the FLAG:CRN8 and EV:GFP treatment. To determine if these changes were due to protein accumulation, we extracted proteins 2 days after infiltration and examined protein levels by Western blot (Figure 6C, E). Loading controls were visualized by Coomassie stain (Figures 6D, F) and indicated equal loading of all protein samples. Figure 6C shows a great reduction in FLAG:CRN8 protein levels when co-expressed with GFP:CRN8^R469A;D470A^, relative to the FLAG:CRN8 levels present when co-expressed with EV:GFP. We attribute this reduction to the presence of the GFP:CRN8^R469A;D470A^ mutant protein because when FLAG:CRN8 is co-expressed with wild type GFP:CRN8, the protein levels remain unaltered (Figure 6C, E). In addition, despite substantially lower protein levels of GFP:CRN8^R469A;D470A^ compared to GFP:CRN8 (Figure 6E), the destabilization impact of the GFP:CRN8^R469A;D470A^ protein on FLAG:CRN8 is greater. Our data indicate that suppression of CRN8-induced cell death is probably due to a reduction in FLAG:CRN8 protein levels, suggesting that the GFP:CRN8^R469A;D470A^ protein destabilizes the FLAG:CRN8 protein.

### CRN8 dimerizes *in planta*

**Figure 7:**
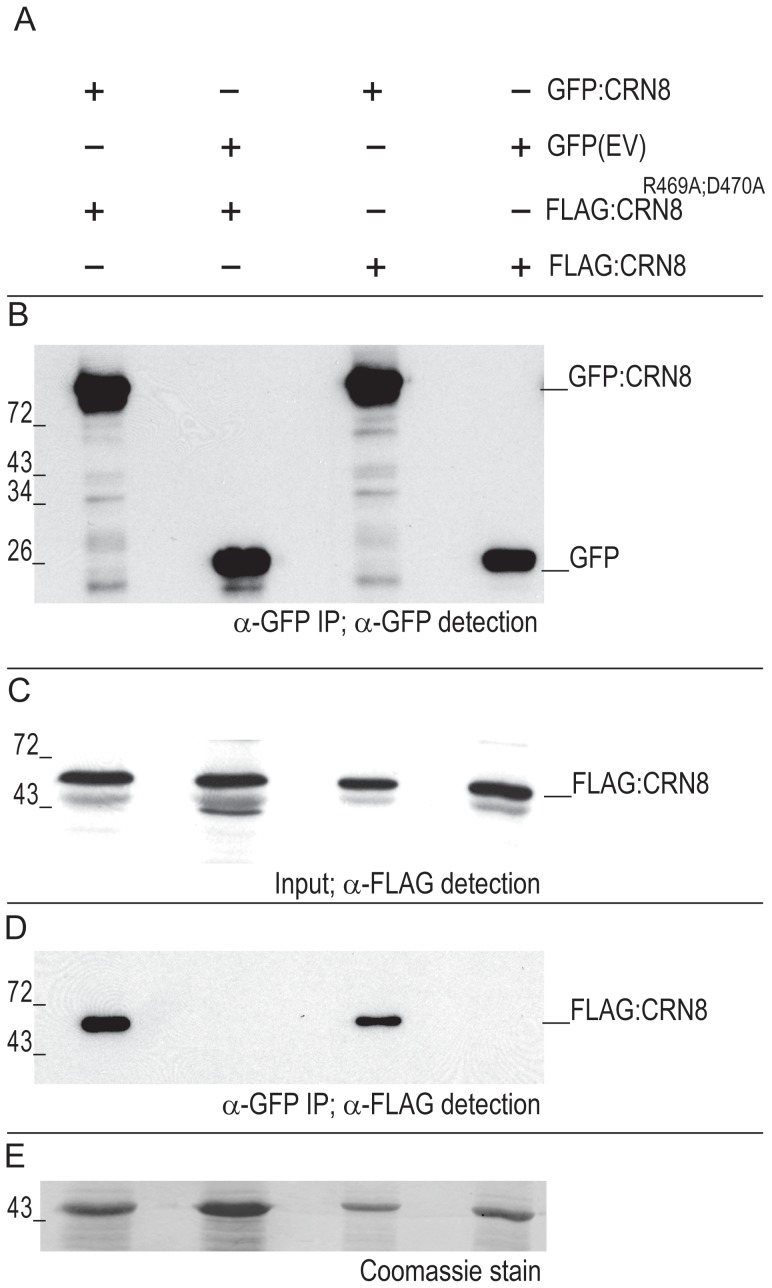
CRN8 forms a dimer. (A) Overview of the different combinations of FLAG:CRN8, GFP:CRN8, FLAG:CRN8^R469A;D470A^, and GFP (indicated by +) proteins, co-expressed in *N. benthamiana* leaves. (B) Western blot of GFP:CRN8 and GFP protein from GFP immuno-purified protein samples probed with GFP antibody. (C) Western blot of FLAG:CRN8 and FLAG:CRN8^R469A;D470A^ inputs for GFP co-immunopurification experiment probed with FLAG antibody. (D) Co-immunoprecipitation of FLAG:CRN8 and FLAG:CRN8 ^R469A;D470A^ in GFP immuno-purified protein samples on a Western blot probed with FLAG antibody. (E) Coomassie stain indicating equal loading of protein on Western blot in figure 7D.

Kinases are known to form dimers [45], and the destabilization CRN8 by CRN8^R469A;D470A^ suggests that these proteins may interact with one another. To test if these proteins dimerize, we co-expressed GFP- and FLAG-tagged CRN8 with FLAG-tagged CRN8^R469A;D470A^ fusion proteins *in planta* and performed co-immunoprecipitation experiments. Figure 7A describes the four different co-expressed protein combinations, including two negative controls. Protein expression of all constructs was verified by Western blot prior to immunoprecipitation for FLAG-tagged proteins and post immunoprecipitation for GFP-tagged proteins (Figure 7B, C). After immunoprecipitation with α-GFP, we observed that only samples containing the GFP:CRN8 fusion (lanes 1 and 3) were able to pull down FLAG:CRN8 or FLAG: CRN8^R469A;D470A^ proteins (Figure 7D), indicating that CRN8 proteins associate *in planta* in a specific manner. The PVDF membrane used for detection of FLAG-tagged protein input was stained with Coomassie blue to verify that proteins were present in relatively equal amounts in all four lanes (Figure 7E). From this experimental data, we conclude that CRN8 occurs as a dimer *in planta* and that dimerization is not impaired in the CRN8^R469A;D470A^ mutant.

### *In planta* expression of CRN8 enhances *P. infestans* virulence, whereas the dominant-negative mutant CRN8^R469A;D470A^ reduces virulence

**Figure 8:**
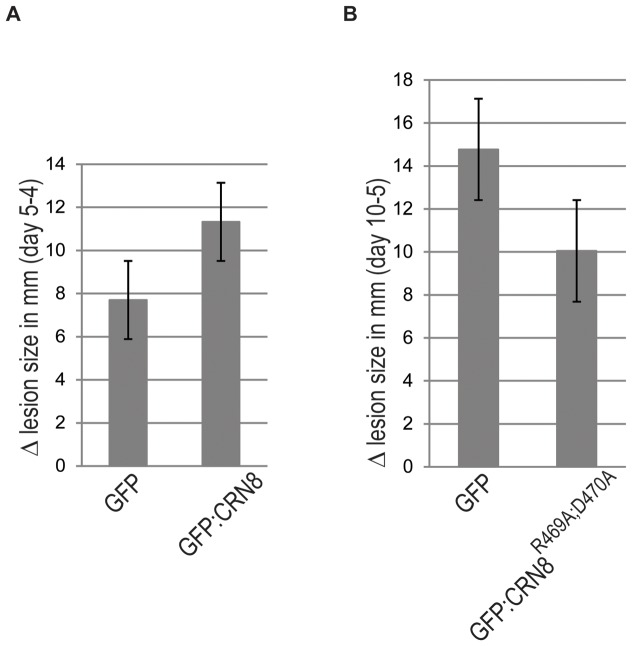
CRN8 is important for full virulence and CRN8^R469A;D470A^ reduces *P. infestans* virulence. (A) The graph shows the growth rate of *P. infestans* lesions when co-inoculated with GFP:CRN8 or GFP. The Y-axis shows the millimeter change in average lesion diameter (n = 21) between days 4 and 5, while the X-axis shows which construct was infiltrated 2 days post *P. infestans* inoculation. Errors bars indicate the standard error (p<0.1). (B) The graph shows the growth rate of *P. infestans* lesions when co-inoculated with GFP:CRN8^R469A;D470A^ or GFP. The Y-axis shows the millimeter change in average lesion diameter (n = 22) between days 5 and 10 post infection, while the X-axis shows which construct was infiltrated before *P. infestans* inoculation. Errors bars indicate the standard error (p<0.0005).

Effectors are thought to play an important role in pathogenicity and plant immunity. To test the extent to which the CRN8 effector increases virulence, we transiently over-expressed both active and inactive kinases, CRN8 and CRN8^R469A;D470A^, in *P. infestans*-challenged *N. benthamiana* leaves. Two days after inoculation with *P. infestans* zoospores, infected *N. benthamiana* leaf panels were infiltrated with *A. tumefaciens* expressing either GFP:CRN8 or EV:GFP. The lesion diameter indicative of pathogen spread was measured 4 and 5 days after *P. infestans* infection, prior to the onset of CRN8-induced cell death (5 dpi). The graph in figure 8A shows an increased rate of *P. infestans* lesion size in the presence of the GFP:CRN8 fusion protein relative to lesions occurring with the negative EV:GFP control. From this pathogenicity assay, we conclude that CRN8 enhances virulence of *P. infestans*.

We also expressed the dominant-negative mutant GFP:CRN8^R469A;D470A^ in *N. benthamiana* and challenged the leaves with *P. infestans* 24 hours after *A. tumefaciens* infiltration. The lesion size was measured 5 and 10 days after *P. infestans* inoculation. Figure 8B shows that the kinase-inactive GFP: CRN8^R469A;D470A^ caused a decrease in *P. infestans* lesion growth rate when compared to the negative control EV:GFP. Given that GFP:CRN8^R469A;D470A^ destabilizes CRN8, it is possible that the decreased virulence we observed is caused by a reduction of the *P. infestans* secreted wild type CRN8 driven by the expression of the dominant-negative CRN8^R469A;D470A^.

## Discussion

In this study, we functionally characterize the CRN8 effector, a secreted kinase from *P. infestans* previously shown to be delivered into plant cells via its N-terminal targeting motif [42]. The *P. infestans* genome consists of gene-dense regions composed mainly of core ortholog genes, and gene-sparse regions which contain many fast-evolving pathogenicity effector families such as the CRNs [9]. The location of the CRN genes in these gene-sparse dynamic regions has likely allowed for the accumulation of rapid evolutionary changes and the considerable expansion of subsets of the CRNs, as evidenced by CRN8 and its homologs. The occurrence of 10 predicted CRN8 paralogs and many additional pseudogenes, all of which carry a highly conserved D2 kinase domain, suggests that the kinase activity of CRN8 is important for *P. infestans* CRN proteins are modular, and recombination of different domains generates an array of chimeric proteins [9]. Our phylogenetic analyses revealed that the CRN8 C-terminus, the kinase domain, has evolved separately from its N-terminal domains. Additionally, this D2 kinase domain segregates into two separate phylogenetic clusters which correspond to the different *Phytophthora* species, *P. infestans* and *P. ramorum* (Figure 1B). This suggests that the expansion of the CRN8 family in the *P. infestans* lineage occurred after its divergence from *P. ramorum*. Indeed, the *P. infestans* paralogs carry only 11 non-synonymous polymorphisms in the D2 kinase domain, a level of polymorphism consistent with recent duplication.

We discovered that CRN8 is an active kinase capable of triggering cell death even in the absence of an intact catalytic site. With the exception of the CRN8^R469A;D470A^ double mutant, mutations that disrupted kinase activity did not alter cell death induction. Previously, we found that cell death induction by the CRN8 protein depends on its nuclear localization [42]. The absence of CRN8 cell death induction by the CRN8^R469A;D470A^ is not due to its mislocalisation. In Figure S3 we show that YFP tagged CRN8^R469A;D470A^ still localizes to the plant nucleus. Therefore, disruption of the kinase activity does not interfere with CRN8 nuclear localization. Interestingly, we were able to show that CRN8^R469A;D470A^ displayed a dominant negative phenotype. Co-expression of CRN8^R469A;D470A^ with CRN8 resulted in a significant reduction of CRN8-induced cell death (Figure 5) and *P. infestans* virulence (Figure 8B). The dominant-negative effect attributed to CRN8^R469A;D470A^ can be accounted for by the destabilization of wild type CRN8 we observed when CRN8^R469A;D470A^ and CRN8 were co-expressed (Figure 6C). Similar antagonistic interference has been noted for other kinases [50], [51], such as the mammalian Ser/Thr kinase, Akt, an important regulator of cell survival and cell proliferation [52], [53]. Co-expression of an inactive Akt kinase mutant inhibited both cell proliferation and apoptopic response in tumor cells expressing the active Akt kinase [46]. Dominant-negative effects have also been documented for plant pathogen effectors, e.g. the effect of truncated derivatives HopM1 to the HopM1 effector [54]. *In planta* expression of the tyrosine phosphatase HopAO1, a Type III secreted effector of the bacterium *P. syringae*, increases bacterial virulence. Expression of the catalytically inactive form, HopAO1^C378S^, interferes specifically with the function of wild type HopAO1 delivered by *P. syringae* by reducing its virulence [13].

We observed that *in planta* expression of CRN8, prior to the onset of cell death, increased virulence of *P. infestans* in *N. benthamiana* (Figure 8A). *P. infestans* is a hemibiotroph that colonizes living plant cells before proliferating on dead plant tissue. Our experiment suggests that CRN8 may contribute to virulence during the biotrophic phase. In addition to CRN8 and other CRNs, several bacterial Type III secretion system effectors and RXLR effectors are known to trigger tissue necrosis, browning, and chlorosis when ectopically expressed in plant cells [9], [39], [55]. The biological relevance of nonspecific cell death promotion by these effectors remains unclear as discussed by Cunnac et al., 2009 and Oh et al. 2009 [39], [55]. The promotion of cell death was proposed to reflect an excessive virulence activity on one or more effector targets [39], [55]. This view is consistent with the finding that CRN8 can enhance virulence prior to the appearance of visible cell death symptoms.

The finding that heterologous expression of CRN8^R469A;D470A^ reduces *P. infestans* virulence raises the possibility of using this mutant to engineer enhanced resistance to late blight in potato and tomato. Heterologous expression of CRN8 and its various mutants in *Solanum lycopersicum*, tomato (Figure S2) a host of *P. infestans* showed similar results of cell death induction as was shown by heterologous expression of CRN8 and the CRN8 mutants in *N. benthamiana*. By creating plants that constitutively express the dominant negative form of CRN8, invading *P. infestans* strains that rely on the delivery of active CRN8 may be unable to effectively colonize their host. Our *P. infestans* virulence assays, show a promising restriction in pathogen spread when CRN8^R469A;D470A^ is expressed *in planta*. Nonetheless, it remains to be determined whether these results will translate into enhanced resistance under field conditions.

Many questions remain about to the mode of action of CRN8 during infection. Does CRN8 act as a kinase, as a substrate, or as part of a complex, or possibly all of the above? CRN8 is likely to auto-phosphorylate, but which plant proteins are targeted and trans-phosphorylated by CRN8? In addition, CRN8 could target and activate plant kinases by forming a complex independent of CRN8 trans-phosphorylation. In the future, identification of CRN8 plant targets will result in a better understanding of how this effector contributes to virulence.

We cannot formally rule out that plant kinases are present in our CRN8 kinase detection assays (Figure 3, Figure 4 and Figure S2). However, this is unlikely because mutations in the active sites R469 and D470 abolished the detected kinase activity. We have also subjected the CRN8 immunoprecipitates, both complete mixture and excised gel fragments, to LC-MS/MS for protein composition and detection of secondary modifications and failed to identify additional proteins. Given that we have not identified other phosphorylated proteins by LC-MS/MS we conclude that the measured kinase activity is mainly due to CRN8.

We identified kinase-like proteins with similarity to CRN8 in the genomes of Arabidopsis, grape, poplar, and several other plant species (MvD and SK, unpublished). Given that kinases, including CRN8, often form dimers and heterodimers, it is possible that CRN8 heterodimerizes with plant kinases [45], influencing their proper activation and subsequent cellular activities. CRN8 might also mimic these plant kinases and target the substrates of the CRN8-like plant kinases. Kinases with closely-related catalytic domains tend to be similar in overall structural topology, have similar modes of regulation, and have similar substrate specificities [45]. Plant pathogen effectors that mimic host proteins are well-known [5]. One example of pathogen mimicry of a plant protein is the bacterial effector AvrPtoB, which is thought to mimic a substrate of a conserved plant kinase, leading to the enhanced activity of host plant kinases [56]. Another example is the secreted kinase from the animal parasitic bacterium *Yersinia pestis*, YpkA [22], [57]. YpkA possesses a domain that binds to the small GTPases RhoA and Rac1. This YpkA domain mimics host guanidine nucleotide dissociation inhibitors (GDIs) for the Rho family of small GTPases and as such, *Yersinia pestis* utilize the Rho GTPases for unique activities during their interaction [23].

To our knowledge, we describe the first plant pathogen effector, CRN8, that encodes a functional kinase domain. The next challenge is to determine the mechanism by which CRN8 functions inside plant cells, which plant process does it ‘hijack’ to interfere with plant immunity. Our study is the first step towards understanding how this secreted kinase effector perturbs the complex kinase signaling network that modulates plant immunity.

## Materials and Methods

### Cloning procedures and plasmid constructs

The different C-and N-terminal deletion mutants were generated by PCR amplification using full length CRN8 as a template with various forward primers that included a *ClaI* restriction site and reverse primers with a *NotI* restriction site. PCR products were digested with ClaI and NotI and were ligated into the *A. tumefaciens* binary potato virus X (PVX) vector pGR106 [58]. The TMV-based expression constructs were generated by amplifying CRN8 variants with the *PacI* restriction site and FLAG sequence embedded in the forward primer and the NotI restriction site included in the reverser primer. These amplicons were then site-directionally cloned into the pTRBO vector [59]. Ligation reactions of pGR106 and pTRBO constructs were directly transformed into *A. tumefaciens* GV3101 or 1D1249 by electroporation. The GFP fused clones were constructed by cloning CRN8 amplicons into the pENTR/D-TOPO (Invitrogen) entry vector followed by Gateway LR recombination (Invitrogen) into pK_7_WGF_2_ [60], resulting in GFP fused CRN8 clones. The different amino acid mutants were generated using primers that introduced the mutation by site directed PCR. For all generated constructs, primers sequences are in Table S2 and all generated sequences were verified to exclude errors.

### NLS prediction

NLS sequence prediction of the CRN8 proteins was done by NLStradamus [61] with a prediction cut off value of 0.6. This approach uses hidden Markov models (HMMs) to predict novel NLSs in proteins (http://www.moseslab.csb.utoronto.ca/NLStradamus/).

### Transient *in planta* protein expression

*In planta* transient expression by Agro-infiltration (*A. tumefaciens* T-DNA 35S promoter based binary constructs) or Agro-infection (PVX or TMV-based binary constructs) was performed according to methods described elsewhere [36], [59], [62] *A. tumefaciens* GV3101 [63] was used to deliver T-DNA constructs into 3-week-old *N. benthamiana* plants. Overnight *A. tumefaciens* cultures were harvested by centrifugation at 10,000 g, resuspended in infiltration medium [10 mM MgCl_2_, 5 mM 2-(N-morpholine)-ethanesulfonic acid (MES), pH 5.3, and 150 mM acetosyringone] to an OD^600^ = 0.3 prior to syringe infiltration into either the entire leaf or leaf sections. For experiments in which co-expression of two constructs was performed in equal ratios, each construct had an OD^600^ = 0.6. In experiments where different co-inoculation ratios were tested, the total amount of each PVX expression combination was OD^600^ = 0.6 of *A. tumefaciens*. The tested PVX ratios included 1∶5; 2∶3 and 1∶1 of *CRN8* with *CRN8^R469A;D470A^* or ratios of 1∶5; 2∶3 and 1∶1 of *CRN8* with *GFP*.

### Protein extractions

Proteins were transiently expressed by *A. tumefaciens* in *N. benthamiana* leaves and harvested two days post infiltration. Immunoblot analyses were performed on protein extracts prepared by grinding leaf samples in liquid nitrogen and extracting in protein extraction buffer [1 gram in 3 ml extraction buffer (150 mM Tris-HCl pH 7.5; 150 mM NaCl; 10% glycerol; 10 mM EDTA; and freshly added 20 mM NaF: 10 mM DTT; 0.5% (w/v) PVPP; 1% (v/v) protease inhibitor cocktail (Sigma); 1% (v/v) NP-40)]. Suspensions were mixed and centrifuged at 5000 rpm for 15 minutes at 4°C. The supernatant was passed through a through 0.45 µm filter before loading.

### Immunoblot analyses

Protein samples (25 µl) were separated by SDS-PAGE (12%) and analyzed by Western blot. PVDF membranes were incubated and washed between different incubation steps with TBS-T (20 mM Tris-HCl, pH 7,5 150 mM NaCl+0.1% Tween). Monoclonal α-FLAG M2 antibody (Sigma-Aldrich) was used as a primary antibody at 1∶8000 (in 5% milk), and anti-mouse antibody conjugated to horseradish peroxidase (HRP, Sigma-Aldrich) was used as a secondary antibody at a 1∶20,000 dilution. For GFP immunoblots, monoclonal α-GFP (Invitrogen) was used as a primary antibody at 1∶4000, and anti-rabbit polyclonal antibody conjugated to horseradish peroxidase (HRP, Sigma-Aldrich) was used as a secondary antibody (1∶12,000 dilution). Blots were developed using the Pierce Horseradish Peroxidase detection kit (Thermo Scientific) and exposed for 2 min on Amersham Hyperfilm ECL (GE Healthcare). Blots were stained with Coomassie (Instant Blue, Expedeon) to visualize protein loading.

### Protein purification

Proteins were extracted from plant material as described above and immuno-purified by FLAG or GFP affinity chromatography. For FLAG immuno-purification: 2.0 ml of extracted protein was incubated with 50 µl anti-FLAG M2 affinity matrix (Sigma) and rotated for 1.5 hr at 4°C followed by 5× wash (centrifuge 30 seconds at 800× g) with 1 ml 50 mM Tris/HCl. Proteins were eluted with 100 µl IP buffer containing 3 µl of 3xFLAG peptide (150 ng/µl), in 97 µl 50 mM Tris/HCl for 30 minutes, shaking gently at 4°C. Samples were centrifuged for 1 minute at 16,000× g and supernatants were saved for either kinase assays or in gel analysis. For the GFP immuno-purification: 1.5 ml of extracted protein were incubated with 20 µl GFP affinity matrix (Chromotek) and rotated for 4 hr at 4°C followed by 5× wash (1 ml TBS+0.5% NP-40) and centrifugation (0.5× g) to pellet beads. 40 µl of 1× Laemmli sample buffer was added and samples were denatured for 5 minutes at a 95°C boil.

### Kinase assays

45 µl of immunoprecipitated protein sample was shaken at 900 rpm for 30 minutes at 27°C with kinase assay buffer [∼185 kBq γ-P^32^-ATP; 20 µM ATP; 50 mM Tris/HCl (pH 7.5); 10 mM MgCl_2_; 10 mM MnCL_2_; and 1 mM DTT]. The reaction was stopped by the addition of 4× Laemmli sample buffer (15 µl) supplemented with 70 mM DTT, and was heated for 5 minutes at 95°C prior to protein separation (25 µl) on an SDS denaturing gel (12%; at 100 V). Similar steps were followed when Pro-Q Diamond phosphoprotein gel stain [49] was used for phosphorylation detection, however in those instances γ-P^32^-ATP was left out of the kinase assay buffer.

### Phosphorylation detection

Detection was done directly on the SDS denaturing gel by Pro-Q Diamond phosphoprotein gel stain [49], or directly after transfer of the separated proteins onto PVDF membrane according to standard Western blotting procedures. Detection of the radio-active phosphorylation signal on the membrane and in the gel was done using the Fuji FLA 5100 phosphor imaging system.

### Phosphorylated peptide detection

Preparation of peptides for liquid chromatography–tandem mass spectrometry (LC-MS/MS) was performed as follows. Proteins were separated with SDS/PAGE. CRN8 fused protein bands were excised from the gel. Gel slices were prepared for LC-MS/MS as described previously [64]. Mass spectrometry LC-MS/MS analysis was performed using a LTQ-Orbitrap mass spectrometer (Thermo Scientific) and a nanoflow-HPLC system (nanoAcquity, Waters Corp.) as described previously [64]. The MS data were searched with Mascot v2.3 (Matrix Science) with the following differences: (i) The database was a custom collection of translated sequences from transcript assemblies (TIGR Plant Transcript Assemblies; http://plantta.jcvi.org) of solanaceous plants (*Solanum lycopersicum*, *N. benthamiana*, *Nicotiana tabacum*, *Solanum tuberosum*, *Capsicum annuum*, and *Petunia hybrida*) and *Phytophthora infestans* sequences containing 1,000,691 sequences (98,308,278 amino acid residues) with the inclusion of sequences of common contaminants such as keratins and trypsin [11]. Carbiodomethylation of cysteine residues was specified as a fixed modification and oxidized methionine and phosphorylation of serine or threonine residues were allowed as variable modifications. Other Mascot (version 2.3) parameters: mass values were monoisotopic and the protein mass unrestricted, the peptide mass tolerance was 5 ppm, and the fragment mass tolerance: ±0.6 Da, two missed cleavages were allowed with trypsin. A second Mascot search was performed allowing ‘error tolerant’ modification of all robustly identified proteins from the previous round. All Mascot searches were collated and verified using Scaffold (Proteome Software) and the subset database was searched using X! Tandem (The Global Proteome Machine Organization Proteomics Database and Open Source Software; www.thegpm.org). Accepted proteins passed the following threshold in Scaffold: 95% protein confidence, with minimum of two unique peptides at 95% confidence.

### Cell death suppression assay

The C-termini of CRN8 (WT) and CRN8^R469A;D470A^ were expressed via the *A. tumefaciens* binary Potato virus X (PVX) vector pGR106 in *N. benthamiana* [36]. *A. tumefaciens* solutions were mixed in 5 different ratios before infiltration into *N. benthamiana* leaves. On the left half of the leaf we co-infiltrated CRN8 (WT) with a truncated GFP construct as a negative control, and on the right half of the leaf we co-infiltrated the *A. tumefaciens* strains containing CRN8 (WT) and the CRN8^R469A;D470A^. To rule out possible concentration effects of the expressed proteins, we tested five different ratios of *A. tumefaciens* concentrations on multiple leaves and varied the position of the expression zones on each leaf. In addition, we used untagged proteins to exclude the possible interference of tags. Cell death phenotypes were scored five days post infiltration.

### Pathogenicity assays

*P. infestans* infection assays were performed by droplet inoculations of zoospore solutions of the *P. infestans* isolate 88069 (10 µl of a 50,000 zoospores per mL solution) on detached *N. benthamiana* leaves. At least ten independent *N. benthamiana* leaves (4 weeks old) were tested per construct combination. For the GFP:CRN8 and the GFP (negative control) comparison, the leaves were challenged with *P. infestans* two days prior to *A. tumefaciens*-mediated expression of these constructs. For the GFP: CRN8^R469A;D470A^ versus the GFP (negative control) comparison, proteins were first expressed by *A. tumefaciens* in *N. benthamiana* 24 hours *prior* to *P. infestans* infection. *P. infestans* growth efficiency was quantified by measuring the lesion size (mm) at 4 and 5 days post infection for the GFP:CRN8 versus the GFP (negative control) expressing leaves. And for GFP:CRN8^R469A;D470A^ versus GFP-expressing leaves, *P. infestans* lesion size (mm) was measured 5 and 10 days after inoculation. Only successful infections at each *Phytophthora* inoculated spot were used to analyze the growth efficiency. The assay was repeated at least three times, and a representative dataset is used.

## Supporting Information

Dataset S1 **Peptide spectra of CRN8 protein.** Peptide spectra of all identified phosphorylated serines by Mass Spectometry in the CRN8 protein.
(ZIP)

Figure S1 **ClustalW alignment of the D2 domain CRN8sequences.** The RD motif is indicated by the 2 asterisks above and the predicted NLS motif (amino acid sequence KGVRKKHRRA) is indicated by a line above the D2 amino acid sequence alignment [42], [61].
(PDF)

Figure S2 **CRN8 cell death induction in tomato.** The top panel shows a cartoon of the CRN8 protein with the RD motif indicated by the two red lines and the five phosphorylated serines indicated as green lines. Asterisks indicate serines that were included in the CRN8^S3xA^ triple mutant. FLAG sequence (blue portion) and the linker sequence (orange portion) are included. The bottom panel shows macroscopic cell death 8 days post infiltration of FLAG:CRN8, FLAG:CRN8^D470N^, FLAG:CRN8^R469A;D470A^, FLAG:CRN8^S3xA^, and FLAG:CRN8^S5xA^ in tomato leaves.
(TIF)

Figure S3 ***In planta*** **nuclear localization of CRN8 and CRN8^R469A;D470A^.** (A) Confocal image of GFP:CRN8 nuclear localization. (B) Confocal image of YFP: CRN8^R469A;D470A^ nuclear localization. (C) Image representing daylight and confocal channel of GFP:CRN8 nuclear localization. (D) Image representing daylight and confocal channel of YFP: CRN8^R469A;D470A^ nuclear localization. (E) Image representing daylight channel of GFP:CRN8. (F) Image representing daylight channel of YFP: CRN8^R469A;D470A^. Scale bar indicates 100 µm. Images of *N. benthamiana* leaves were taken 3 days post infiltration.
(TIF)

Table S1 **List of phosphorylated peptides.** List of amino acid sequences of phosphorylated peptides that were identified in multiple experiments.
(XLSX)

Table S2 **Primers used for the presented CRN8 study.**
(PDF)

Text S1 **Materials and methods for Figure S2 and S3.**
(DOCX)
