# Correction: The Irish Potato Famine Pathogen *Phytophthora infestans* Translocates the CRN8 Kinase into Host Plant Cells

**DOI:** 10.1371/journal.ppat.1004753

**Published:** 2015-03-25

**Authors:** The PLOS Pathogens Staff

There is an error in Fig. 2. This figure contains two duplicated leaf panels. The authors have provided a corrected version of the figure here.

**Fig 2:**
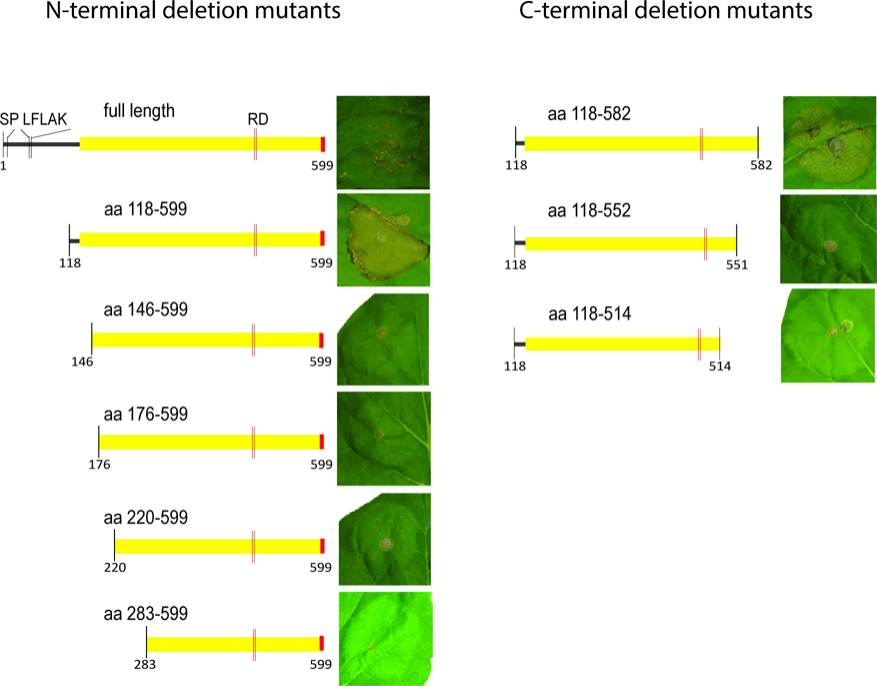
Defining the CRN8 cell death induction domain. *In planta* expression of various N-terminal or C-terminal deletions of CRN8, demonstrating the minimal domain necessary for cell death induction. The D2 domain is indicated in yellow, whereas the red portion indicates the position of the functional NLS.
