# Supplementary figures and images for: The Irish Potato Famine Pathogen Phytophthora infestans Translocates the CRN8 Kinase into Host Plant Cells
Source: PLoS Pathog. 2012 Aug 23;8(8):e1002875. doi: 10.1371/journal.ppat.1002875 (PMC3426532; doi:10.1371/journal.ppat.1002875)

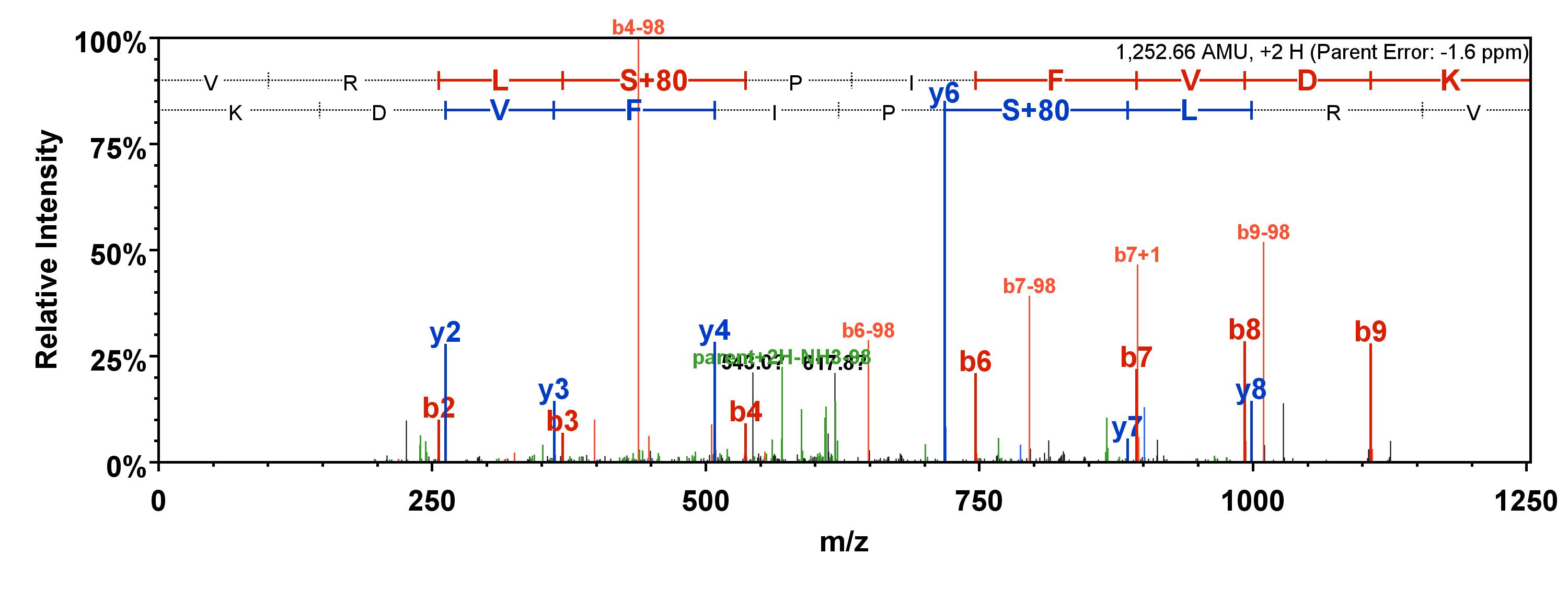

Supplement: Dataset S1 — Peptide spectra of CRN8 protein. Peptide spectra of all identified phosphorylated serines by Mass Spectometry in the CRN8 protein. (ZIP) [file ppat.1002875.s001.zip › SPECTRA/2_VRLsPIFVDK.jpg]

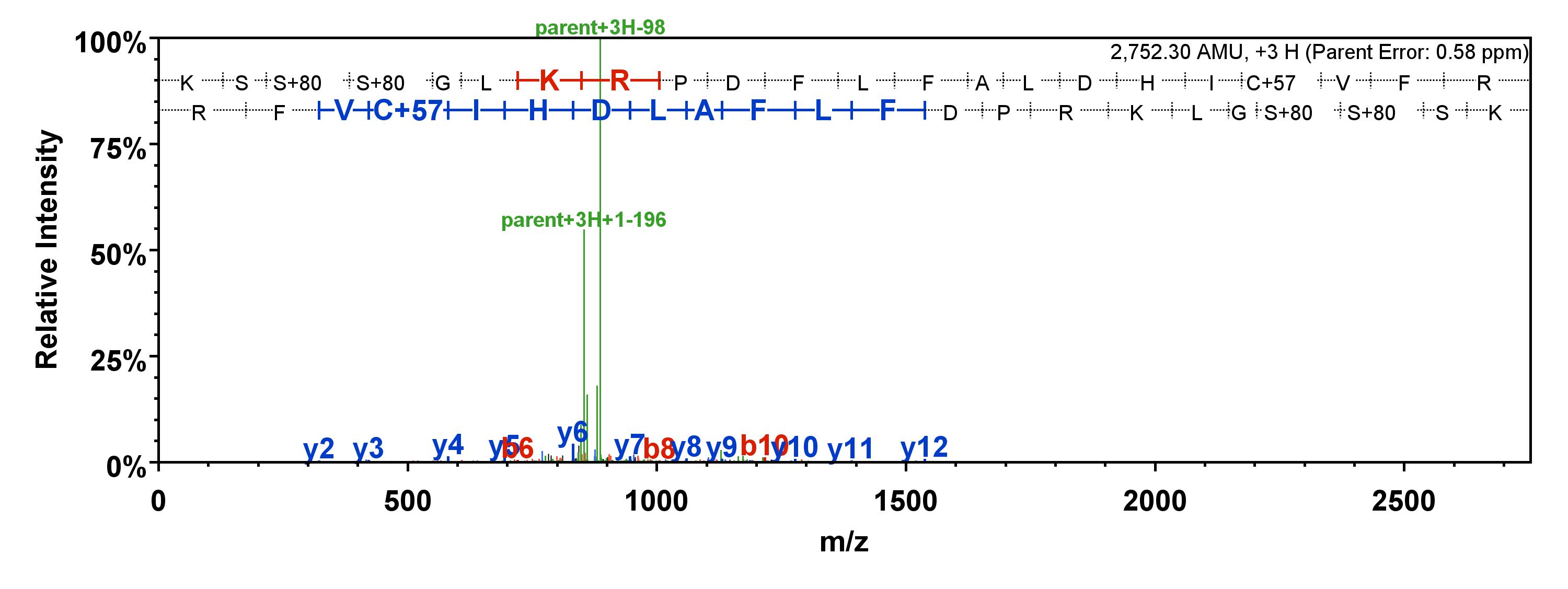

Supplement: Dataset S1 — Peptide spectra of CRN8 protein. Peptide spectra of all identified phosphorylated serines by Mass Spectometry in the CRN8 protein. (ZIP) [file ppat.1002875.s001.zip › SPECTRA/2P_KSssGLKRPDFLFALDHIcVFR.jpg]

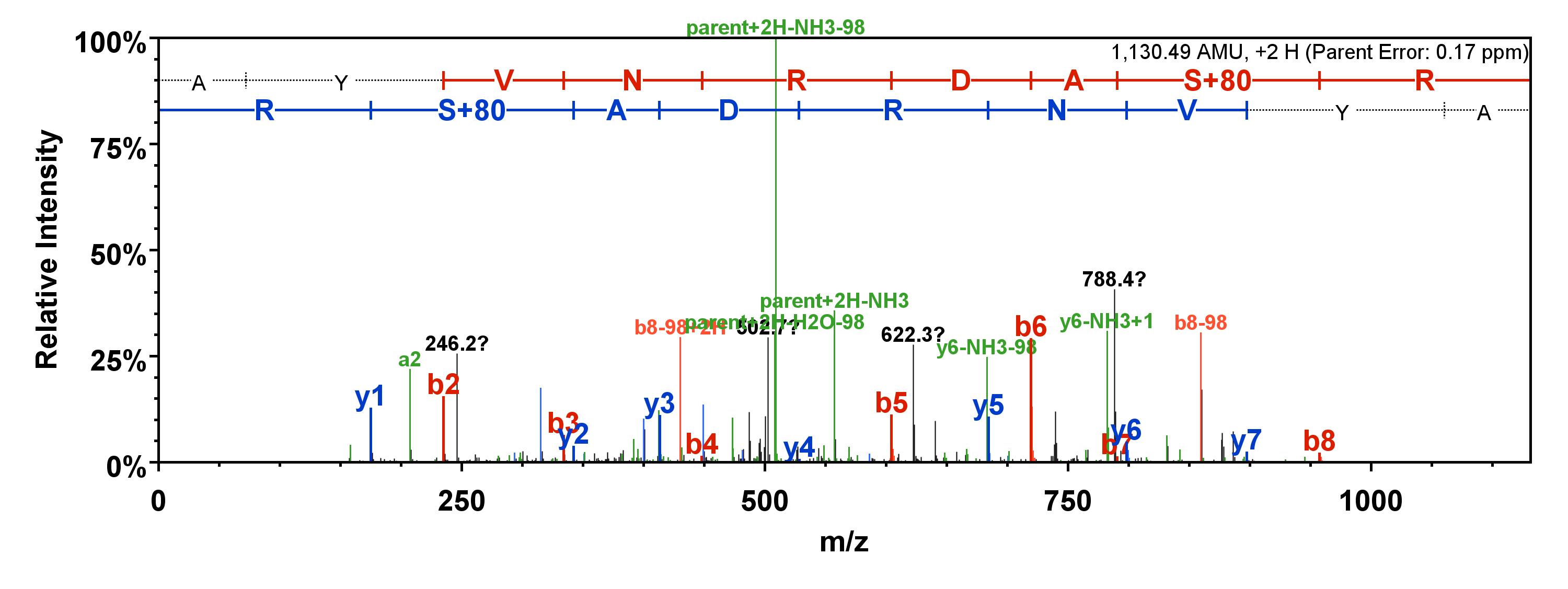

Supplement: Dataset S1 — Peptide spectra of CRN8 protein. Peptide spectra of all identified phosphorylated serines by Mass Spectometry in the CRN8 protein. (ZIP) [file ppat.1002875.s001.zip › SPECTRA/AYVNRDASR.jpg]

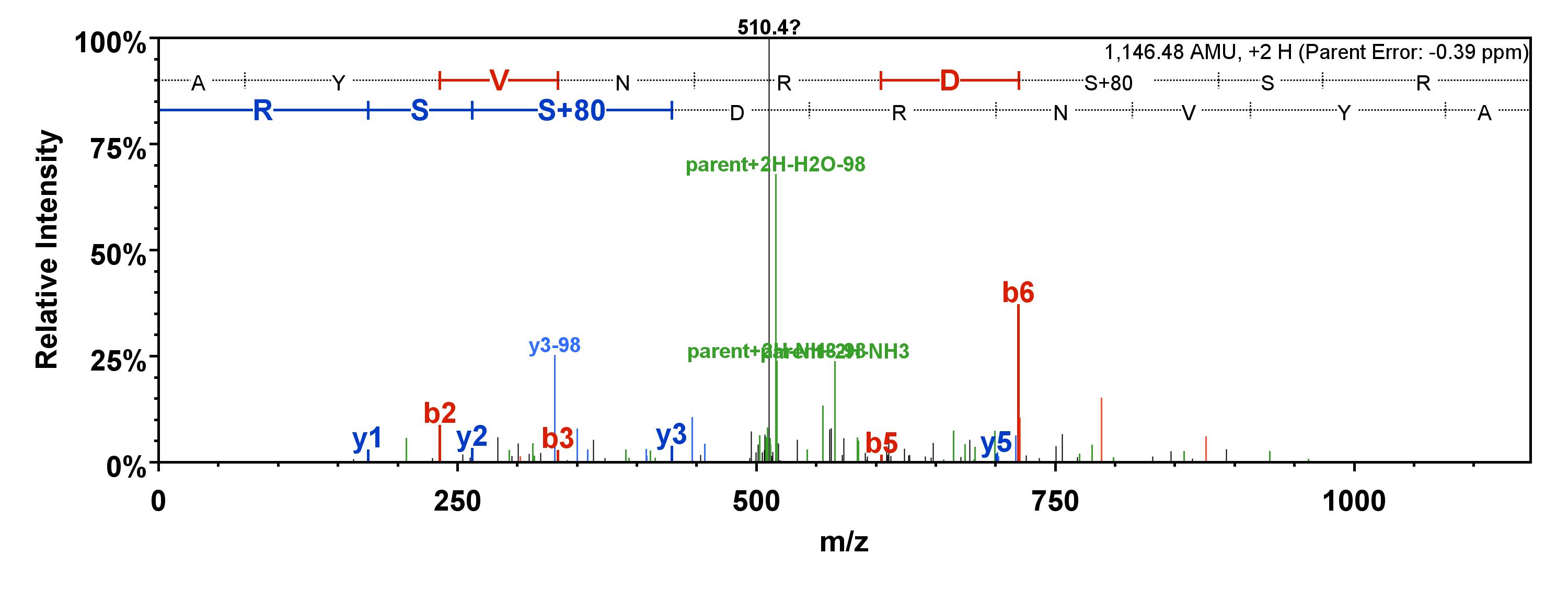

Supplement: Dataset S1 — Peptide spectra of CRN8 protein. Peptide spectra of all identified phosphorylated serines by Mass Spectometry in the CRN8 protein. (ZIP) [file ppat.1002875.s001.zip › SPECTRA/AYVNRDSSR.jpg]

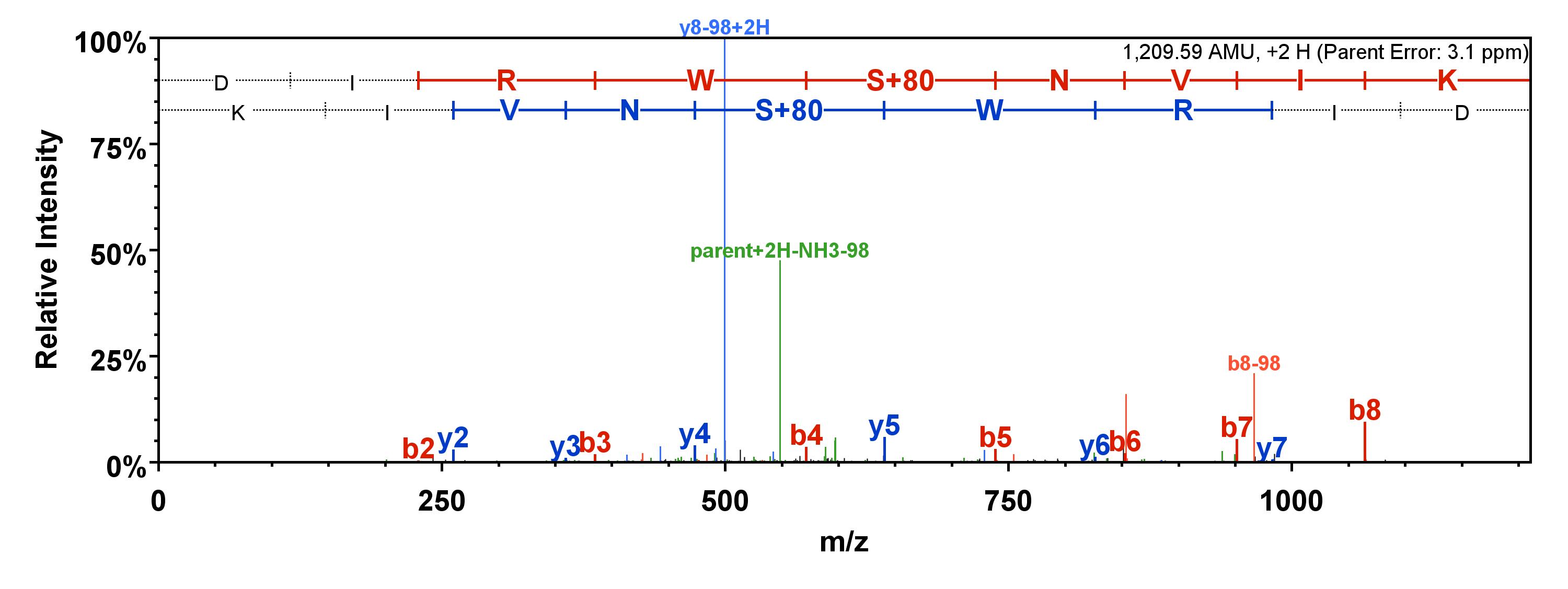

Supplement: Dataset S1 — Peptide spectra of CRN8 protein. Peptide spectra of all identified phosphorylated serines by Mass Spectometry in the CRN8 protein. (ZIP) [file ppat.1002875.s001.zip › SPECTRA/DIRWsNVIK.jpg]

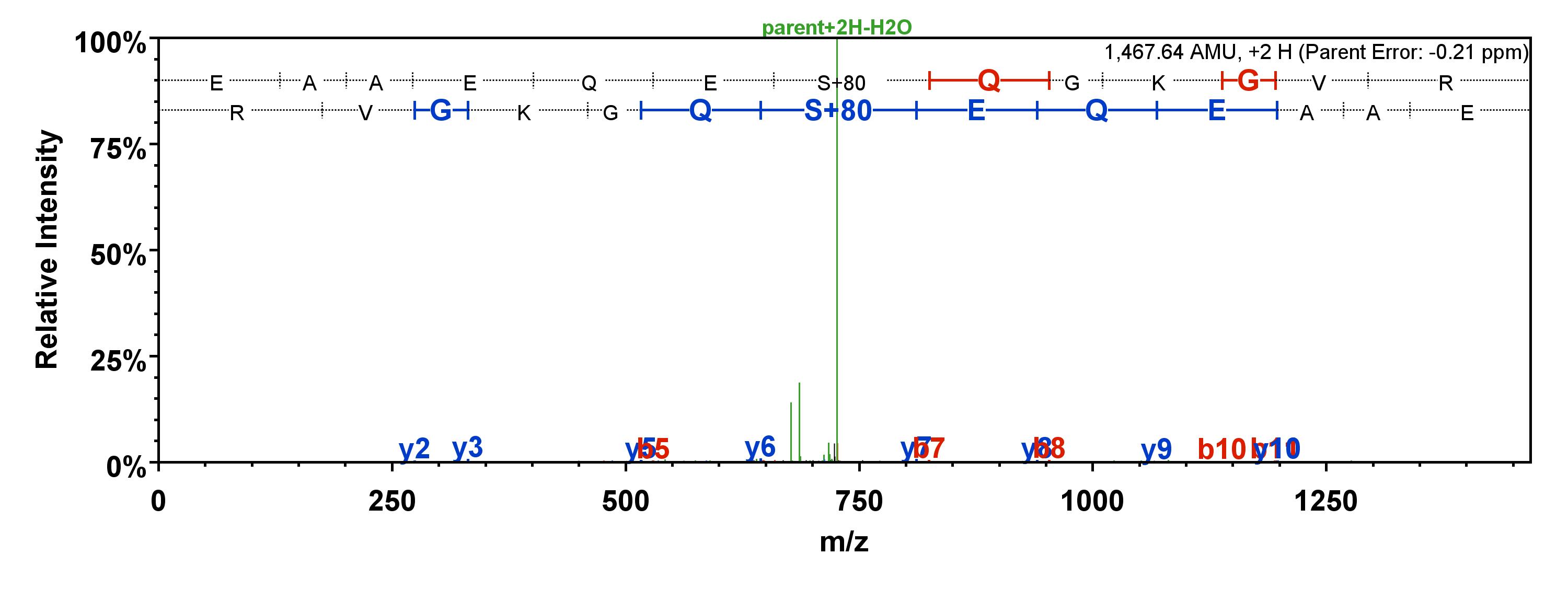

Supplement: Dataset S1 — Peptide spectra of CRN8 protein. Peptide spectra of all identified phosphorylated serines by Mass Spectometry in the CRN8 protein. (ZIP) [file ppat.1002875.s001.zip › SPECTRA/EAAEQEsQGKGVR.jpg]

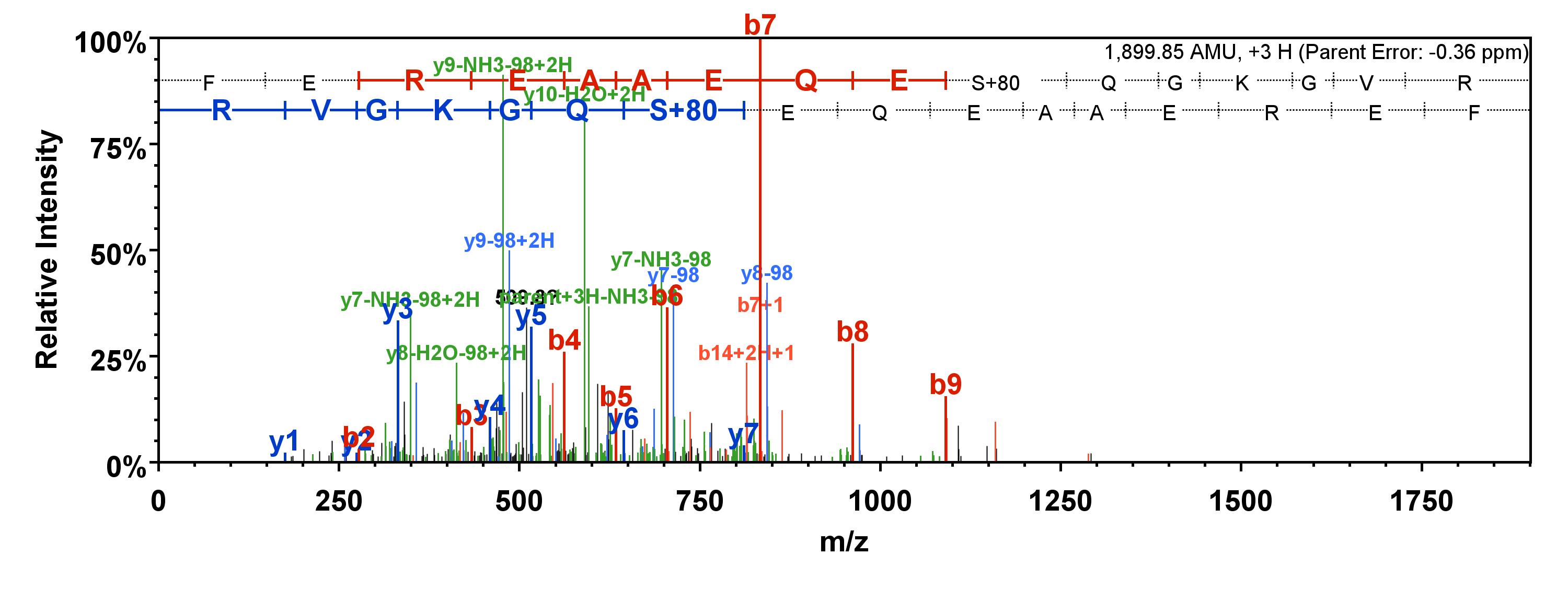

Supplement: Dataset S1 — Peptide spectra of CRN8 protein. Peptide spectra of all identified phosphorylated serines by Mass Spectometry in the CRN8 protein. (ZIP) [file ppat.1002875.s001.zip › SPECTRA/FEREAAEQEsQGKGVR.jpg]

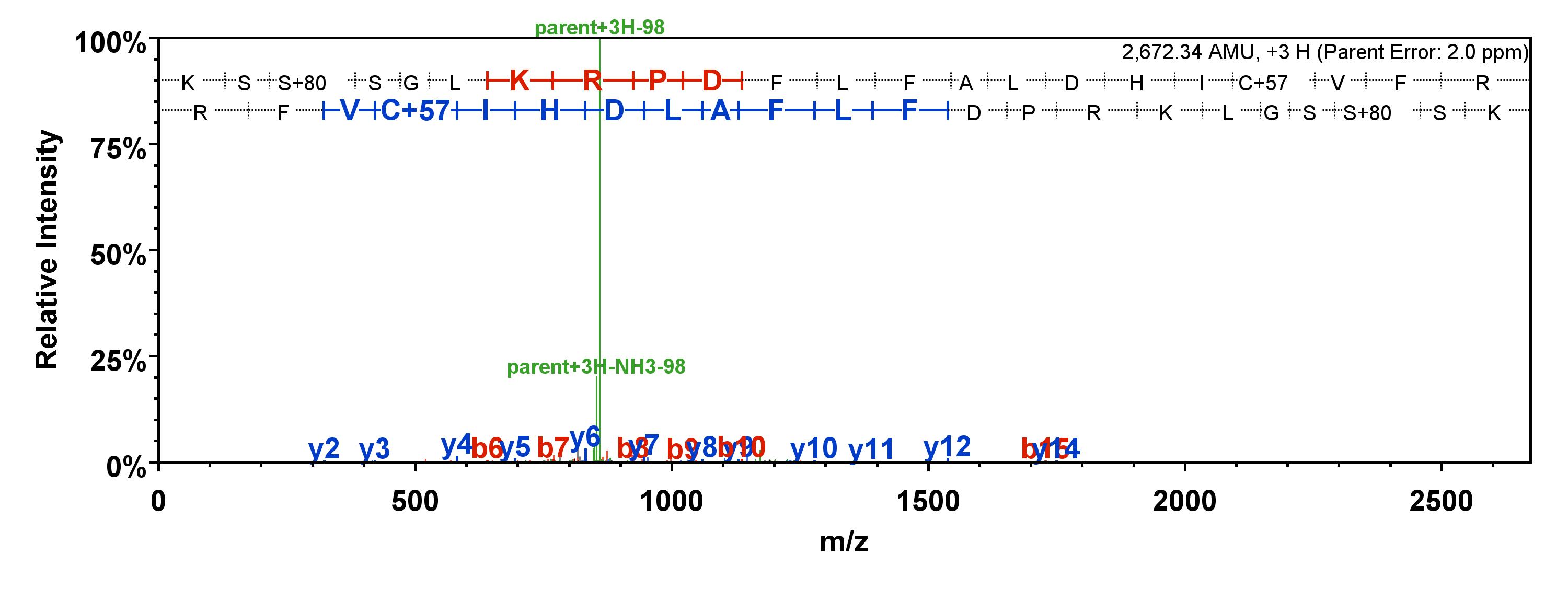

Supplement: Dataset S1 — Peptide spectra of CRN8 protein. Peptide spectra of all identified phosphorylated serines by Mass Spectometry in the CRN8 protein. (ZIP) [file ppat.1002875.s001.zip › SPECTRA/KSsSGLKRPDFLFALDHIcVFR.jpg]

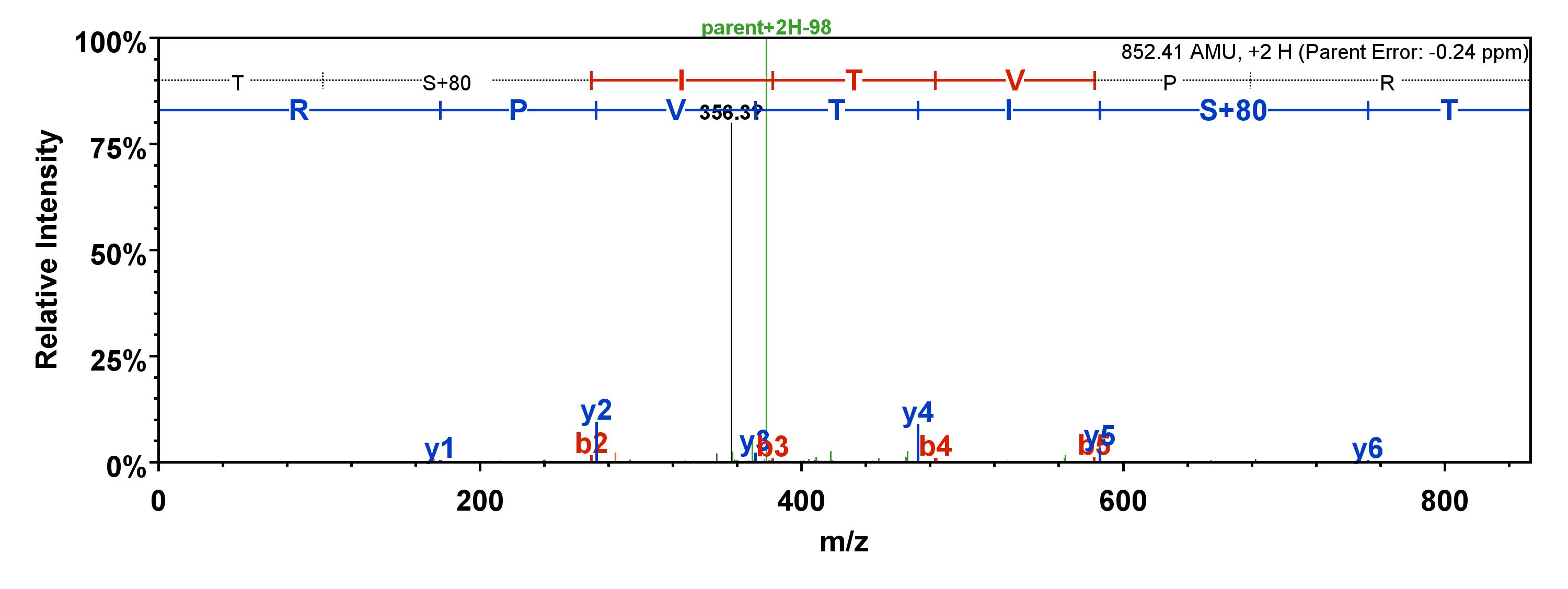

Supplement: Dataset S1 — Peptide spectra of CRN8 protein. Peptide spectra of all identified phosphorylated serines by Mass Spectometry in the CRN8 protein. (ZIP) [file ppat.1002875.s001.zip › SPECTRA/TSITVPR.jpg]

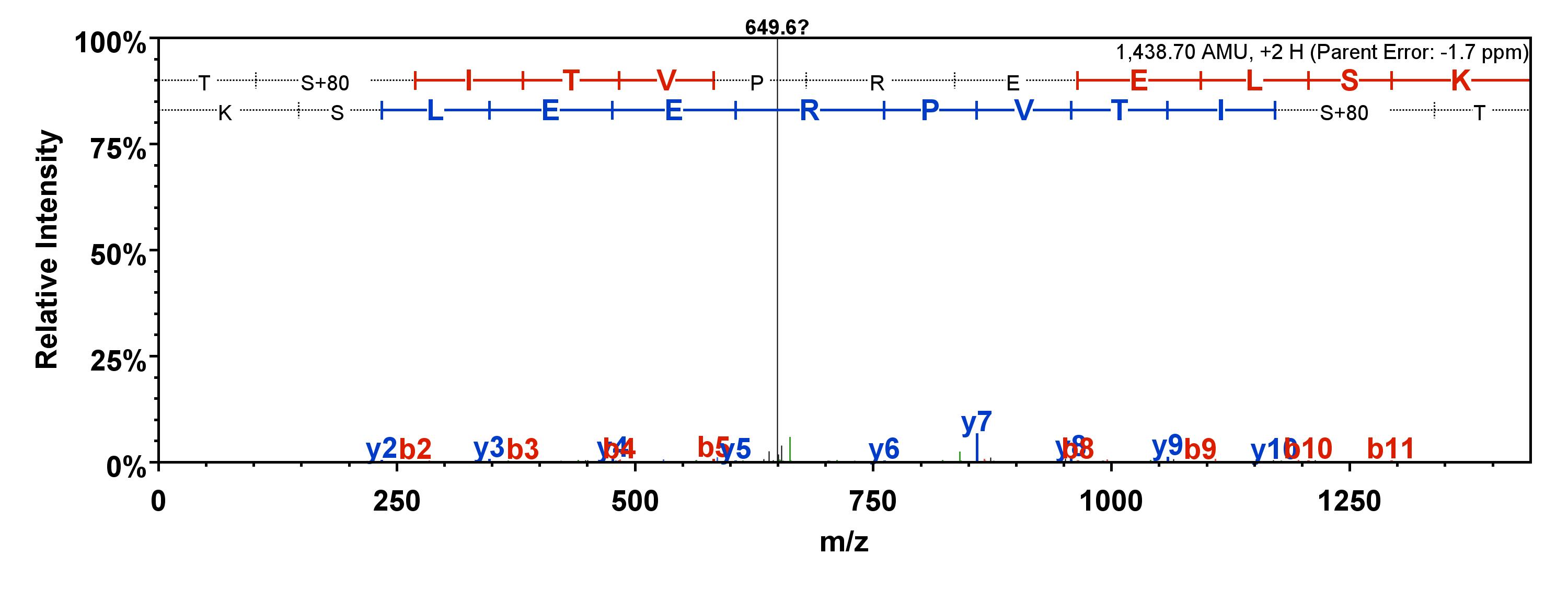

Supplement: Dataset S1 — Peptide spectra of CRN8 protein. Peptide spectra of all identified phosphorylated serines by Mass Spectometry in the CRN8 protein. (ZIP) [file ppat.1002875.s001.zip › SPECTRA/TSITVPREELSK.jpg]

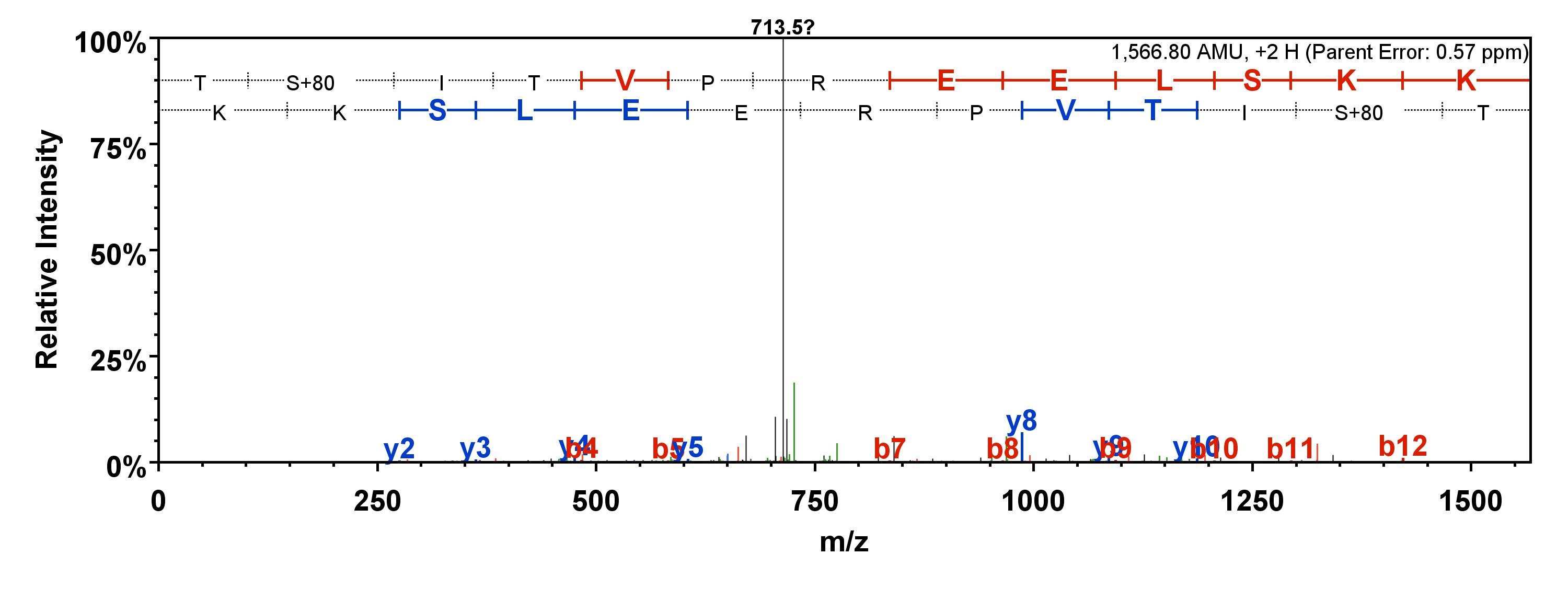

Supplement: Dataset S1 — Peptide spectra of CRN8 protein. Peptide spectra of all identified phosphorylated serines by Mass Spectometry in the CRN8 protein. (ZIP) [file ppat.1002875.s001.zip › SPECTRA/TSITVPREELSKK.jpg]

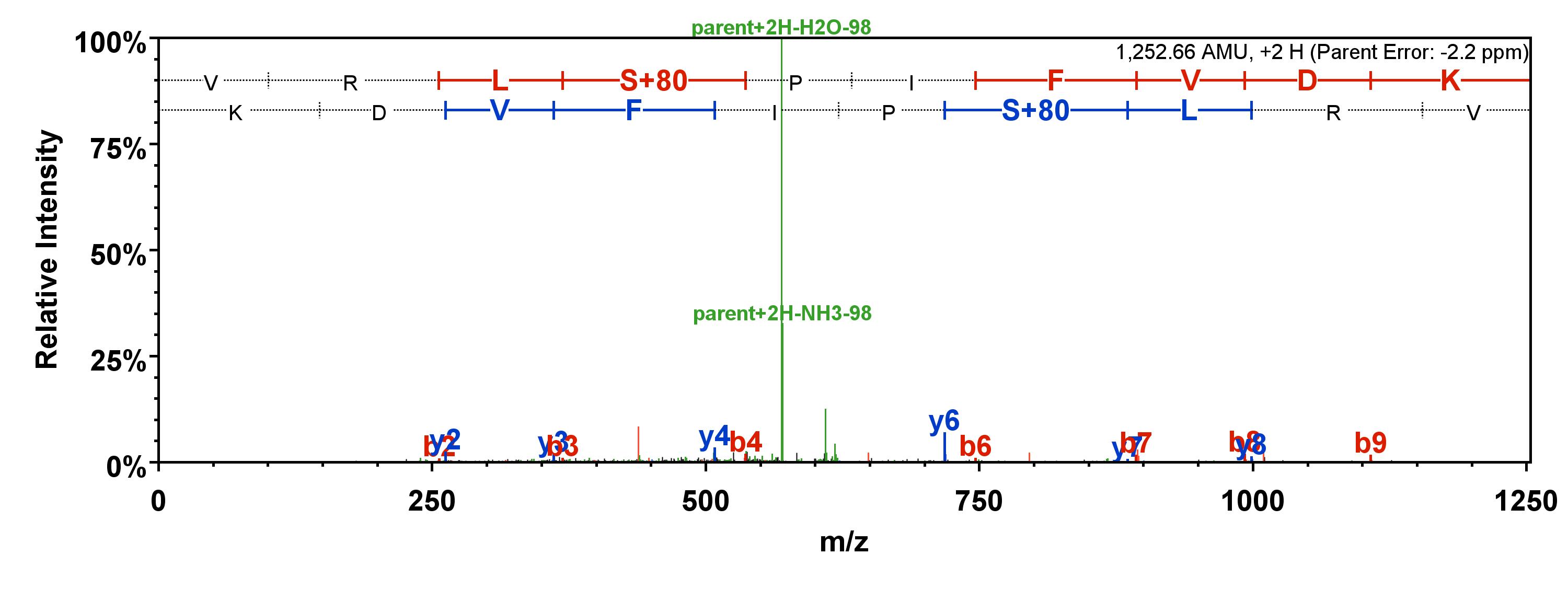

Supplement: Dataset S1 — Peptide spectra of CRN8 protein. Peptide spectra of all identified phosphorylated serines by Mass Spectometry in the CRN8 protein. (ZIP) [file ppat.1002875.s001.zip › SPECTRA/VRLSPIFVDK.jpg]

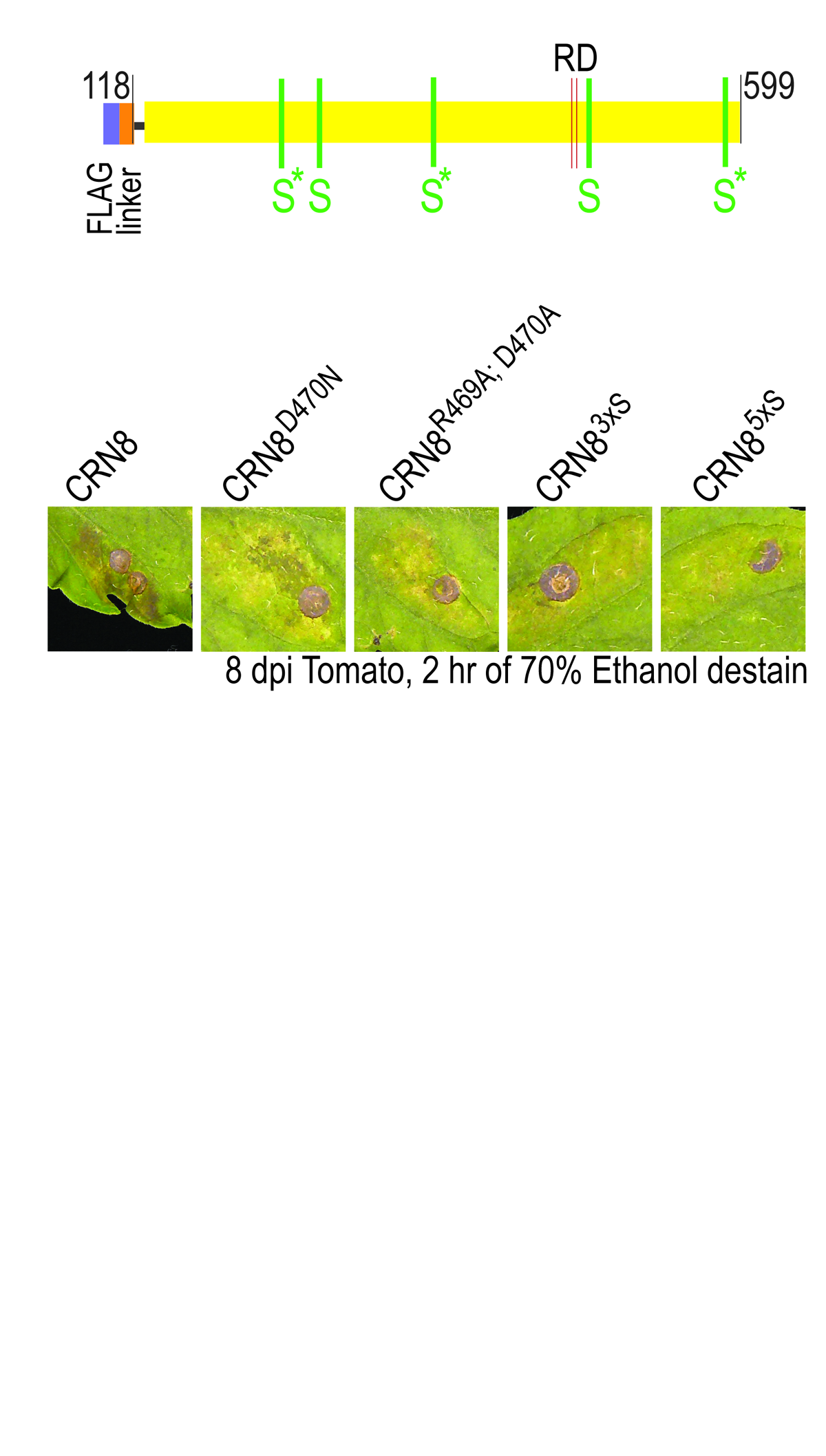

Supplement: Figure S2 — CRN8 cell death induction in tomato. The top panel shows a cartoon of the CRN8 protein with the RD motif indicated by the two red lines and the five phosphorylated serines indicated as green lines. Asterisks indicate serines that were included in the CRN8S3xA triple mutant. FLAG sequence (blue portion) and the linker sequence (orange portion) are included. The bottom panel shows macroscopic cell death 8 days post infiltration of FLAG:CRN8, FLAG:CRN8D470N, FLAG:CRN8R469A;D470A, FLAG:CRN8S3xA, and FLAG:CRN8S5xA in tomato leaves. (TIF) [file ppat.1002875.s003.tif]

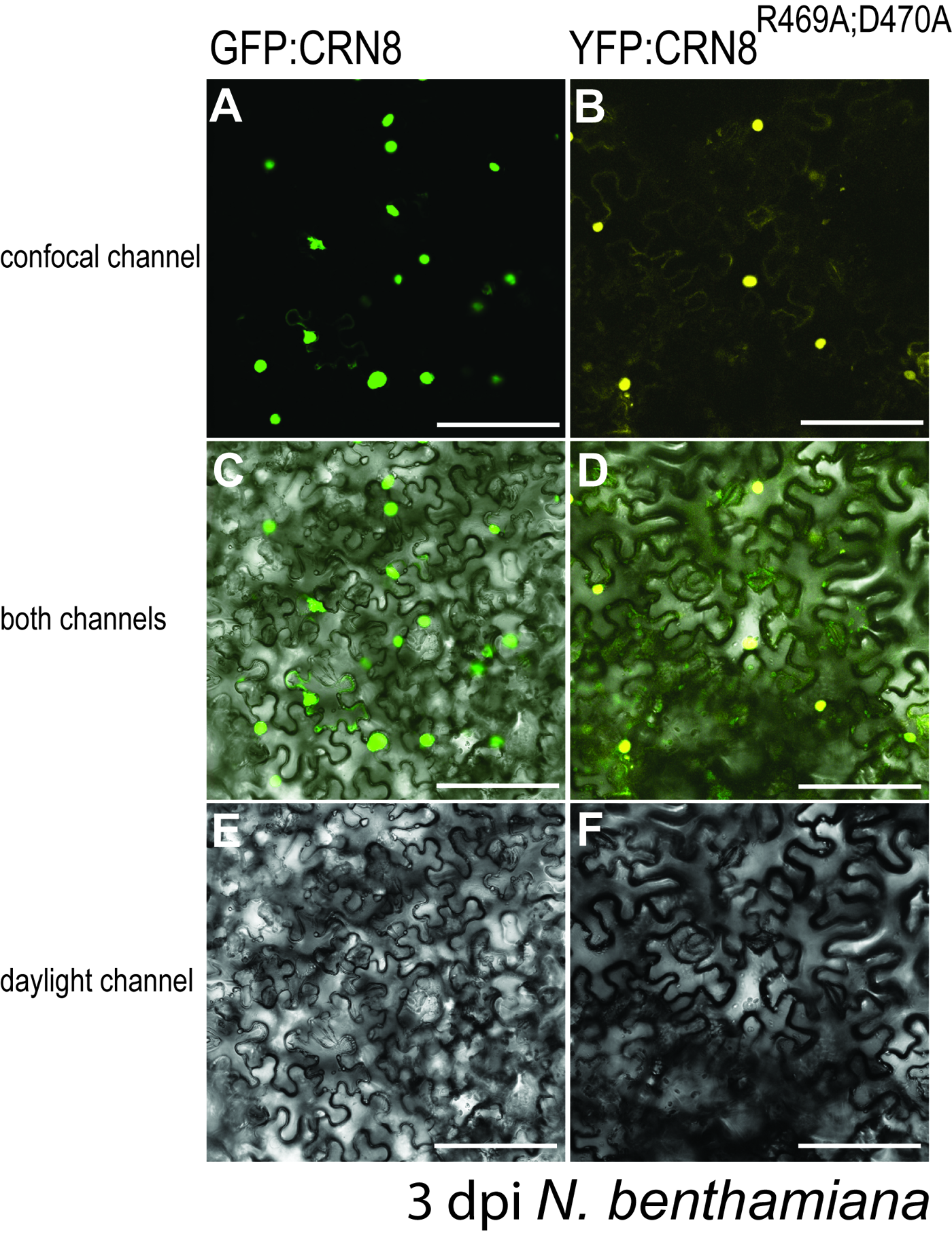

Supplement: Figure S3 — In planta nuclear localization of CRN8 and CRN8R469A;D470A. (A) Confocal image of GFP:CRN8 nuclear localization. (B) Confocal image of YFP: CRN8R469A;D470A nuclear localization. (C) Image representing daylight and confocal channel of GFP:CRN8 nuclear localization. (D) Image representing daylight and confocal channel of YFP: CRN8R469A;D470A nuclear localization. (E) Image representing daylight channel of GFP:CRN8. (F) Image representing daylight channel of YFP: CRN8R469A;D470A. Scale bar indicates 100 µm. Images of N. benthamiana leaves were taken 3 days post infiltration. (TIF) [file ppat.1002875.s004.tif]
