# Supplementary material for: The Irish Potato Famine Pathogen Phytophthora infestans Translocates the CRN8 Kinase into Host Plant Cells
Source: PLoS Pathog. 2012 Aug 23;8(8):e1002875. doi: 10.1371/journal.ppat.1002875 (PMC3426532; doi:10.1371/journal.ppat.1002875)
Supplement: Figure S1 — ClustalW alignment of the D2 domain CRN8sequences. The RD motif is indicated by the 2 asterisks above and the predicted NLS motif (amino acid sequence KGVRKKHRRA) is indicated by a line above the D2 amino acid sequence alignment [42], [61]. (PDF) [file ppat.1002875.s002.pdf]

```
Pi_19589    1 DLWLSRFQHSEVAKLTLLPTRGDLNEFIGQPLPVKIGLPQSVFQAWSSPLILGQL--LRD
Pi_12090    1 DLWLSRFQHSEVAKLTLLPTRGDLNEFIGQPLPVKIGLPQSVFQAWSSPSILGQL--LRD
Pi_23274    1 DLWLSRFQHSEVAKLTLLPTRGDLNEFIGQPLPVKIGLPQSVFQAWSSPLILGQL--LRD
Pi_12094    1 DLWLSRFQHSEVAKLTLLPTRGDLNEFIGQPLPVKIGLPQSVFQAWSSPLILGQL--LRD
Pi_16585    1 DLWLSRFQHSEVAKLTLLPTRGDLNEFIGQPLPVKIGLPQSVFQAWSSPSILGQL--LRD
Pi_16618    1 DLWLSRFQHSEVAKLTLLPTRVDLNEFIGQPLPVKIGLPQSVFQAWSSPSILGQL--LRD
Pi_19318    1 DLWLSRFQHCEVAKLTLLPTRGDLNEFIGQPLPVKIGLPQSVFQAWSSPLILGQL--LRD
Pi_12646    1 DLWLSRFQHCEVAKLTLLPTRGDLNEFIGQPLPVKIGLPQSVFQAWSSPLILGQL--LRD
Pi_16636    1 DLWLSRFQHSEVAKLTLLPTRGDLNEFIGQPLPVKIGLPQSVFQAWSSPLILGQL--LRD
CRN8        1 DLWLSRFQHSEVAKLTLLPTRGDLNEFIGQPLPVKIGLPQSVFQAWSSPLILGQL--LRD
Pi_19565    1 DLWLSRFQHSEVAKLTLLPTRGDLNEFIGQPLPVKIGLPQSVFQAWSSPLILGQL--LRD
Pr_84114    1 DAWIKAIKDEQVTELP--STCEDLREHLQRPLHVKVPVNDRLFLIVSTKNTTGELSSILD
Pr_80840    1 DAWIKAIKDEQVTELP--STCEDLREHLQRPLHVKVPVNDRLFLIVSTKNTTGELSSILD
Pr_75879    1 DAWIKAIKDEQVTELP--STCEDLKEHLQRPLHVKVPVNDRLFLIMSTKNTTGELSSILD

Pi_19589   59 KLFELNDISPCEFLKDSVFSAAFLYPQVDG-DATESAFHYFWDSIIRVVLGFVFRRAYVN
Pi_12090   59 KLFELNDISPCEFLKDSVFSAAFLYPQVDG-DATESAFHYFWDSIIRVVLGFVFRRAYVN
Pi_23274   59 KLFELNDISPCEFLKDSVFSAAFLYPQVDG-DATESAFHYFWDSIIRVVLGFVFRRAYVN
Pi_12094   59 KLFELNDISPCEFLKDSVFSAAFLYPQVDG-DATESAFHYFWDSIIRVVLGFVFRRAYVN
Pi_16585   59 KLFELNDISPCEFLKDSVFSAAFLYPQVDG-DATESAFHYFWDSIIRVVLGFVFRRAYIN
Pi_16618   59 KLFELNDISPCEFLKDSVFSAAFLYPQVDG-DATESAFHYFWDSIIRVVLGFVFRRAYIN
Pi_19318   59 KLFELNDISPCEFLKDSVFSAAFLYPQVDG-DATESAFHYFWDSIIRVVLGFVFRRAYIN
Pi_12646   59 KLFELNDISPCDFLKDSVFSAAFLYPQVDG-DATESAFHYFWDSIIRVVLGFVFRRAYVN
Pi_16636   59 KLFELNDISPCEFLKDSVFSAAFLYPQVDG-DATESAFHYFWDSIIRVVLGFVFRRAYVN
CRN8       59 KLFELNDISPCEFLKDSVFSAAFLYPQVDG-DATESAFHYFWDSIIRVVLGFVFRRAYVN
Pi_19565   59 KLFELNDISPCEFLKDSVFSAAFLYPQVDG-DATESAFHYFWDSIIRVVLGFVFRRAYIN
Pr_84114   59 KVFEPDPRETLSDITAGILGSVIDPLGSGSEFATEDTYHHLWDSLIAMLLRRVSN-GKFR
Pr_80840   59 KVFEPDPRETRSDITAGILGSVIDPLGSGSEFATEDTYHHLWDSLIAMLLRRVSN-GKFR
Pr_75879   59 KVFEPDPRKTLSDITAGILGSVIDPLGSGSEFATEDTYHHLWDSLIAMLLRRVSN-GKFR

Pi_19589  118 RDSSRKSSSGLKRPDFLFALD--HICVFRGEEKEPRTSITVPREELSKKLVWSYGGVPYV
Pi_12090  118 RDSSRKSSSGLKRPDFLFALD--HICVFRGEEKEPRTSITVPREELSKKLVWSYGGVPYV
Pi_23274  118 RDSSRKSSSGLKRPDFLFALD--HICVFRGEEKEPRTSITVPREELSKKLVWSYGGVPYV
Pi_12094  118 RDSSRKSLSGLKRPDFLFALD--HICVFRGEEKEPRTSITVPREELSKKLVWSYGGVPYV
Pi_16585  118 RDSSRKSSSGLKRPDFLFALD--HICVFRGEEKEPRTSITVPREELSKTLVWSYGGVPYV
Pi_16618  118 RDSSRKSSSGLKRPDFLFALD--HICVFRGEEKEPRTSITVPREELSKTLVWSYGGVPYV
Pi_19318  118 RDSSRKSSSGLKRPDFLFALD--HICVFRGEEKEPRTSINVPREELSKKLVWSYGGVPYV
Pi_12646  118 RDSSRKSSSGLKRPDFLFALD--HICVFRGEEKEPRTSITVPREELSKKLVWSYGGVPYV
Pi_16636  118 RDSSRKSSSGLKRPDFLFALD--HICVFRGEEKEPRTSITVPREELSKKLVWSYGGVPYV
CRN8      118 RDSSRKSSSGLKRPDFLFALD--HICVFRGEEKEPRTSITVPREELSKKLVWSYGGVPYV
Pi_19565  118 RDSSRKSSSGLKRPDFLFALD--HICVFRGEEKEPRTSITVPREELSKKLVWSYGGVPYV
Pr_84114  118 RNTNASTSTGLYRPDLCFYYKNSNVCVVRGEEQ-ASGELQVPVRELHEKLTWRYDAAPYV
Pr_80840  118 RNTNASTSTDLYRPDLCFYYKNSNVCVVRGEEK-ASGELQVPVRELYEKLTWRYDAAPYV
Pr_75879  118 RNTNASTSTSLYRPDLCFYYKNSNVCVVRGEEK-ASGELQVPVRKLHEKLTWRYDAAPYV

Pi_19589  176 FGYAASGFELELFAIYQDVTGN--VKTHLIGGFNLQHAPERFRLVLALLNLCLLFPAIVQ
Pi_12090  176 FGYAASGFELELFAIYQDVTGN--VKTHLIGGFNLQHAPERFRLVLALLNLCLLFPAIVQ
Pi_23274  176 FGYAASGFELELFAIYQDVTGN--VKTHLIGGFNLQHAPERFRLVLALLNLCLLFPAIVQ
Pi_12094  176 FGYAASGFELELFAIYQDVTGN--VKTHLIGGFNLQHAPERFRLVLALLNLCLLFPAIAQ
Pi_16585  176 FGYAASGFELELFAIYQDVTGN--VKTHLIGGFNLQHAPERFRLVLALLNLCLLFPAIVQ
Pi_16618  176 FGYAASGFELELFAIYQDVTGN--VKTHLIGGFNLQHAPERFRLVLALLNLCLLFPAIVQ
Pi_19318  176 FGYAASGFELELFAIYQDVTGN--VKTHLIGGFNLQHAPERFRLVLALLNLCLLFPAIVQ
Pi_12646  176 FGYAASGFELELFAIYQDVTGN--VKTHLIGGFNLQHAPERFRLVLALLNLCLLFPAIVQ
Pi_16636  176 FGYAASGFELELFAIYQDVTGN--VKTHLIGGFNLQHAPERFRLVLALLNLCLLFPAIVQ
CRN8      176 FGYAASGFELELFAIYQDVTGN--VKTHLIGGFNLQHAPERFRLVLALLNLCLLFPAIVQ
Pi_19565  176 FGYAASGFELELFAIYQDVTGN--VKTHLIGGFNLQHAPERFRLVLALLNLCLLFPAIVQ
Pr_84114  177 FGYAAVGLQVCLVAIRKDEMTERGAKVEIIETYDLGDLNGRLSFFLALLNLSTLFRPVVD
Pr_80840  177 FGYAAVGLQVCLVAIRKDEMTERGAKVEIIETYDLGDLNGRLSFFLALLNLSTLFRPVVD
Pr_75879  177 FGYAAVGLQVCLVAIRKDEMTERGAKVEIIETYDLGDLNGRLSFFLALLNLSTLFRPVVD
```

```
Pi_19589  234 NCPASAGTEFMDIHRANGVKVRLSPIFVDKIFHT----QEEYRRVKRIYDSLKAYRIPCA
Pi_12090  234 NCPASAGTEFIDIHRANGVKVRLSPIFVDKNFHT----QEEYRRVKRIYDSLKAYRIPCA
Pi_23274  234 NCPASAGTEFMDIHRANGVKVRLSPIFVDKNFHT----QEEYRRVKRIYDSLKAYRIPCA
Pi_12094  234 NCPASAGTEFMDIHRANGVKVRLSPIFVDKIFHT----QEEYRRVKRIYDSLKAYRIPCA
Pi_16585  234 NCPASAGAEFMDIHRANGVKVRLSPIFVDKIFHT----QEEYRRVKQIYDSLKAYGVPCA
Pi_16618  234 NCPASAGTEFMDIHRANGVKVRLSPIFVDKIFHT----QEEYRRVKQIYDSLKAYGVPCA
Pi_19318  234 NCPASAGTEFMDIHRANGVKVRLSPIFVDKIFHT----QEEYRRVKQIYDSLKAYGVPCA
Pi_12646  234 NCPASAGTEFMDIHRANGVKVRLSPIFVDKIFHT----QEEYRRVKQIYDSLKAYGVPCA
Pi_16636  234 NCPASAGTEFMDIHRANGVKVRLSPIFVDKIFHT----QEEYRRVKQIYDSLKAYGVPCA
CRN8      234 NCPASAGTEFMDIHRANGVKVRLSPIFVDKIFHT----QEEYRRVKQIYDSLKAYGVPCA
Pi_19565  234 NCPASAGTEFMDIHRANGVKVRLSPILVDKIFHT----QEEYRRVKQIYDSLKAYGVPCA
Pr_84114  237 LIQPLDILEYGTIERDNGVQITFAEDCVVKTYPQNMPSDDIIRNLRELHRQMKKHSVPNV
Pr_80840  237 LIQPLDILEYGTIERGNGVQITFAEDCVVKTYPQNMPSDDIIRNLRELHRQMKKHSVPNV
Pr_75879  237 IIQPLDILEYGTIERGNGVQITFAEDCVVKTYPQNMPSDDIIRNLRELHRQMKKHSVPNV

                                                           **
Pi_19589  290 DAVVTVDSDQLRLTLKPRGVEMKPCSLSELFVALGNVLEALVVLHRNGWMHRDIRWSNVI
Pi_12090  290 DAVVTVDSDQLRLTLKPRGVEMKPCSLSELFVALGNVLEALVVLHRNGWMHRDIRWSNVI
Pi_23274  290 DAVVTVDSDQLRLTLKPRGVEMKPCSLSELFVALGNVLEALVVLHRNGWMHRDIRWSNVI
Pi_12094  290 DAVVTVDSDQLRLTLKPRGVEMKPCSLSELFVALGNVLEALVVLHRNGWMHRDIRWSNVI
Pi_16585  290 DAVVTVDSDQLRLTLKPRGIEMKPCSLSELFVALGNVLEALVVLHRNGWMHRDIRWSNVI
Pi_16618  290 DAVVTVDSDQLRLTLKPRGIEMKPCSLSELFVALGNVLEALVVLHRNGWMHRDIRWSNVI
Pi_19318  290 DAVVTVDSDQLRLTLKPRGIEMKPCSLSELFVALGNVLEALVVLHRNGWMHRDIRWSNVI
Pi_12646  290 DAVVTVDSDQLRLTLKPRGIEMKPCSLSELFVALGNVLEALVVLHRNGWMHRDIRWSNVI
Pi_16636  290 DAVVTVDSDQLRLTLKPRGIEMKPCSLSELFVALGNVLEAPVVLHRNGWMHRDIRWSNVI
CRN8      290 DAVVTVDSDQLRLTLKPRGIEMKPCSLSELFVALGNVLEALVVLHRNGWMHRDIRWSNVI
Pi_19565  290 DAVVTVDSDQLRLTLKPRGIEMKPCSLSELFVALGNVLEALVVLHRNGWMHRDIRWSNVI
Pr_84114  297 VDLKKTNMTKKYVKLAPVGRLSPPVNAHQLLTALRDILQALVALHAINLMHRDLRWENVL
Pr_80840  297 VELKKTNMTKKYVKLAPVGRLSRPENAHQLLTALRDILQALVALHAINLMHRDLRWENVL
Pr_75879  297 VGLKKTNMTKKYVKLAPVGRLSRPENAHQLLTALRDILQALVALHAINLMHRDLRWENVL

Pi_19589  350 KHIDR-VEWFLIDFADAAQSPQKYPSGDHLTHDEHASDIFMEGGSHTTAVDLWAVGYLVK
Pi_12090  350 KHIDR-VEWFLIDFADAAQSPQKYPSGDHLTHDEHASDIFMEGGSHTTAVDLWAVGYLVK
Pi_23274  350 KHIDR-VEWFLIDFADAAQSPQKYPSGDHLTHDEHASDIFMEGGSHTTAVDLWAVGYLVK
Pi_12094  350 KHIDR-VEWFLIDFADAAQSPQKYPSGDHLTHDEHASDIFMEGGSHTTAVDLWAVGYLVK
Pi_16585  350 KHIDR-VEWFLIDFADAAQSPQKYPSGDHLTHDEHASDIFMEGGSHTTAVDLWAVGYLVK
Pi_16618  350 KHIDR-VEWFLIDFADAAQSPQKYPSGDHLTHDEHASDIFMEGGSHTTAVDLWAVGYLVK
Pi_19318  350 KHIDR-VEWFLIDFADAAQSPQKYPSGDHLTHDEHASDIFMEGGSHTTAVDLWAVGYLVK
Pi_12646  350 KHIDR-VEWFLIDFADAAQSPQKYPSGDHLTHDEHASDIFMEGGSHTTAVDLWAVGYLVK
Pi_16636  350 KHIDR-VEWFLIDFADAAQSPQKYPSDDHLTHDEHASDIFMEGGSHTTAVDLWAVGYLVK
CRN8      350 KHIDR-VEWFLIDFADAAQSPQKYPSGDHLTHDEHASDIFMEGGSHTTAVDLWAVGYLVK
Pi_19565  350 KHIDR-VEWFLIDFADAAQSPQKYAQRRSLDARRTRFRYNYGRWIPHDCG----------
Pr_84114  357 KYPGEGDKWFLIDFDEGASSP--AAKVKHLKAESHAPEISSSS--HTVKVDIWSVGFLLM
Pr_80840  357 KYPGEGDKWFLIDFDEGASSP--AAKVKHLKAESHAPEILLSSS-HTVKVDIWSVGFLLM
Pr_75879  357 KYPGEGDKWFLIDFDEGASSP--AAKVKHLKAESHAPEISSSS--HTVKVDIWSVGFLLM

                                                          NLS
Pi_19589  409 TSKIEREWTAEPERALSWIG----------------------------------------
Pi_12090  409 TSKIEREWTAEPERALFLDRLMNTDPSARPTADEALQLLSRFEREAAEQDHRARVCARSK
Pi_23274  409 TSKIEREWTAEPERALFLDRLMNTDPSARPTADEALQLLSRFEREAAEQESQGKGVRKKH
Pi_12094  409 TSKIEREWTAEPERALFLDRLMNTDPSARPTADEALQLLSRFEREAAEQESQGKGVRKKQ
Pi_16585  409 TSKIEREWTAEPERALS-------------------------------------------
Pi_16618  409 TSKIEREWTAEPERALFLDRLMNPDPSARPTADEALQLLSRFEREAAEQESQGKGVRKKH
Pi_19318  409 TSKIEREWTAEPERALFLDRLMNPDPSARPTADEALQLLSRFEREAAEQESQGKGVRKKH
Pi_12646  409 TSKIEREWTAEPERVLFLDRLMNTDPSARPTADEALQLLSRFEREAAEQESQGKGVRKKH
Pi_16636  409 TSKIEREWTAEPERALFLDRLMNPDPSARPTADEALQLLSRFEREAAEQESQGKGVRKKH
CRN8      409 TSKIEREWTAEPERALFLDRLMNPDPSARPTADEALQLLSRFEREAAEQESQGKGVRKKH
Pi_19565      ------------------------------------------------------------
Pr_84114  413 TSPYQ---DLPPELESVKAQCLQKNPSSRPTAESLLKVVESLIES---------------
Pr_80840  414 TSPCQ---DLPPELESVKAQCLQKSPSSRPTAESLLKVVESLIES---------------
Pr_75879  413 TSPCH---DLPPELESVKAQCLQKNPSSRPTAESLLKVVESLIES---------------
```

```
                  ___
Pi_19589          ----------------------
Pi_12090    469   DVREFLHRYGTTITRWDEVLWY
Pi_23274    469   RRA-------------------
Pi_12094    469   RRA-------------------
Pi_16585          ----------------------
Pi_16618    469   RRA-------------------
Pi_19318    469   RRV-------------------
Pi_12646    469   RRA-------------------
Pi_16636    469   RCA-------------------
CRN8        469   RRA-------------------
Pi_19565          ----------------------
Pr_84114          ----------------------
Pr_80840          ----------------------
Pr_75879          ----------------------
```
