# Supplementary material for: The Irish Potato Famine Pathogen Phytophthora infestans Translocates the CRN8 Kinase into Host Plant Cells
Source: PLoS Pathog. 2012 Aug 23;8(8):e1002875. doi: 10.1371/journal.ppat.1002875 (PMC3426532; doi:10.1371/journal.ppat.1002875)
Supplement: Table S2 — Primers used for the presented CRN8 study. (PDF) [file ppat.1002875.s006.pdf]

Supplemental Table 2. Primers for generated constructs and mutations

**PVX cloning (pGR106 ClaI-NotI)**	
CRN8_F1	GCGATCGATGGTTACTTTGTTCTGTGCCGTCGTTGGTGTG
CRN8_F118	GCGCCATGGAATCGATGGGACAAGTAGTGCGCCTATTTCTG
CRN8_F146	GCGCCATGGAATCGATGCTGCCGACACGCGGAGATCTAAATGAATTTATC
CRN8_F176	GCGCCATGGAATCGATGTTGATACTAGGCCAACTCCTTCGAGATAAATTG
CRN8_F220	GCGCCATGGAATCGATGAGTGCATTTCATTATTTCTGGGATTCAATTATC
CRN8_F282	GCGCCATGGAATCGATGATCACTGTGCCTCGTGAGGAGCTCTCTAAAAAG
CRN8_R514	GCGGCGGCCGCTCACGTGTGGGATCCACCTTCCATAAAAATATC
CRN8_R551	GCGGCGGCCGCTCACGCTCTTTCGGGCTCCGCAGTCCATTC
CRN8_582	GCGGCGGCCGCTCAAGCTTCACGTTCAAAACGTGACAGAAGCTGTAAGGC
CRN8_R	GCGCTGCAGCGGCCGCTCAGGCACGTCTGTGCTTCTTGCGCAC

**pENTR**	
CRN8_pENTR_F_MvD	caccATGGGGACAAGTAGTGCGCCTATTTCTG
CRN8 pENTR_R_MvD	TCAGGCACGTCTGTGCTTCTTGCGCACACCC

**pTRBO cloning (pJL48 PacI-NotI)**	
CRN8_118_F_pJL48_MvD	CGATGATGATTCGGAGGCTACTGTCGCCG
CRN8_599_R_pJL48_MvD	GATTACGGACACAATCCGTTATTTATTATGCATCTTG

**RD to AA mutation**	
CRN8_F_AAtoRD_MvD	AACGGGTGGATGCACGCAGCCATCCGTTGGTCTAACGTGAT
CRN8_R_AAtoRD_MvD	TTAGACCAACGGATGGCTGCGTGCATCCACCCGTTGCGA

**D to N mutation**	
F_CRN8_D470N_MvD	CGGGTGGATGCACAGAAACATCCGTTGGTC
R_CRN8_D470N_MvD	GACCAACGGATGTTTCTGTGCATCCACCCG

**S to A mutations**	
F_CRN8_S249A_MvD	CGTCAACCGGGATTCGGCCAGGAAATCC
R_CRN8_S249A_MvD	GGATTTCCTGGCCGAATCCCGGTTGACG
F_CRN8_S281A_MvD	GAACCTCGTACTGCTATCACTGTGCCTCG
R_CRN8_S281A_MvD	CGAGGCACAGTGATAGCAGTACGAGGTTC
F_CRN8_S385A_MvD	GTCAAGGTGCGGTTGGCTCCGATTTTCG
R_CRN8_S385A_MvD	CGAAAATCGGAGCCAACCGCACCTTGAC
F_CRN8_S474A_MvD	CATCCGTTGGGCTAACGTGATCAAGC
R_CRN8_S474A_MvD	GCTTGATCACGTTAGCCCAACGGATG
F_CRN8_S587A_MvD	CGTGAAGCTGCTGAGCAGGAAGCACAGGGC
R_CRN8_S587A_MvD	GCCCTGTGCTTCCTGCTCAGCAGCTTCACG
