# Supplementary material for: The Irish Potato Famine Pathogen Phytophthora infestans Translocates the CRN8 Kinase into Host Plant Cells
Source: PLoS Pathog. 2012 Aug 23;8(8):e1002875. doi: 10.1371/journal.ppat.1002875 (PMC3426532; doi:10.1371/journal.ppat.1002875)
Supplement: Text S1 — Materials and methods for Figure S2 and S3. (DOCX) [file ppat.1002875.s007.docx]

**Material and Methods**

**Transient in planta protein expression**

In planta transient expression by Agro-infection (TMV-based binary constructs) was performed according to methods in the main text. A. tumefaciens 1D1249 (Wroblewski *et al.*, 2005) was used to deliver T-DNA constructs into 4-week-old tomato, Cf-0 Money Maker plants. Overnight A. tumefaciens cultures were harvested by centrifugation at 10,000 g, resuspended in infiltration medium [10 mM MgCl2, 5 mM 2-(N-morpholine)-ethanesulfonic acid (MES), pH 5.3, and 150 mM acetosyringone] to an OD600 = 0.3 prior to syringe infiltration into either the entire leaf or leaf sections.

**Confocal Microscopy.**

Cut leaf patches were mounted in water and analysed on a Leica DM6000B/TCS SP5 confocal microscope (Leica Microsystems) with the GFP, 488 nm excitation wavelength. Scanning was performed in sequential mode to prevent signal bleed-through. Two different microscope power settings were applied for the GFP and the YFP images because the fluorescence intensity of the GFP was stronger than the YFP samples.
